# Species identification skills predict in-depth knowledge about species

**DOI:** 10.1371/journal.pone.0266972

**Published:** 2022-04-18

**Authors:** Michiel Jan Dirk Hooykaas, Menno Schilthuizen, Casper Johannes Albers, Ionica Smeets

**Affiliations:** 1 Science Communication and Society, Leiden University, Leiden, The Netherlands; 2 Naturalis Biodiversity Center, Leiden, The Netherlands; 3 Heymans Institute for Psychological Research, University of Groningen, Groningen, The Netherlands; Oklahoma State University, UNITED STATES

## Abstract

To raise biodiversity awareness effectively, communicators should be aware of knowledge levels in their audiences. Species identification skills have been used in the past as a measure of what people know about species, yet it is not known whether they serve as good indicators. To study the link between species identification and in-depth species knowledge, we presented an animal knowledge test to an online audience of over 7,000 Dutch adults, and used correlation and regression analyses to determine the extent to which species identification predicts in-depth knowledge about species’ origin, habitat, diet, and behavior. We found that in-depth knowledge was higher in those who correctly identified species as compared with those who did not correctly identify species, for all four types of in-depth knowledge. Moreover, as compared to alternative variables (work, age, gender, and educational level), species identification was by far the best predictor for in-depth knowledge about species. However, species identification levels were generally higher than levels of in-depth knowledge, and knowledge gaps and misconceptions were uncovered. The results confirm the value of species identification tests, but also highlight limitations and challenges that should be taken into account when establishing knowledge levels and communicating biodiversity.

## Introduction

Communication plays a vital role in building biodiversity awareness and public support for conservation. To do this effectively, biodiversity communicators should be aware of knowledge levels in their target groups. Prior knowledge influences the way in which audiences respond [1–3], and materials can then be crafted according to existing knowledge gaps and misconceptions. However, research has shown that while people may be aware of their own level of knowledge [4], it is generally quite difficult to estimate knowledge levels in target audiences [5–8]. In different fields, professionals struggle with making accurate judgements, even when they are confident about their estimation and prediction skills [9, 10]. This makes effective ways of assessing prior knowledge in the public highly important. Species identification tests have regularly been used to measure people’s knowledge about species [11–21], and to establish levels of ecological knowledge [22, 23] and knowledge about nature in general [24]. However, empirical proof that identification skills are good indicators of in-depth understanding is lacking.

Species identification skills are an important component of species literacy, a concept coined by Hooykaas et al. [18] that combines both ‘broad knowledge about species’ (notably the ability to identify, i.e. recognize and name species) and ‘in-depth knowledge about species’ (e.g. knowing where species occur, what they eat, and how they behave). Species literacy is regarded as a starting point towards awareness about biodiversity [25], which is crucial for building broad-based support in society for conservation [26–28].

Although levels of both species identification [17, 18] and in-depth species knowledge [29–31] have been reported in the past, few studies have explored broad and in-depth species knowledge simultaneously, and when they did [32, 33], it was not reported how these were associated with each other. While it is often assumed that when people identify species correctly this also reflects their in-depth knowledge about those species, it is not known whether this is indeed the case. On the one hand an association between identification skills and in-depth knowledge is plausible, as recognition and naming can lead people to learn more about a species [34, 35]. Moreover, even though authors have argued that people mainly use anatomical features to identify species [36], people use environmental and behavioral clues too. For example, an elephant on the African savannah will be recognized as an African elephant; a lizard may commonly be distinguished from a newt by noting that the animal is basking in the sun, not swimming underwater. Even names themselves may reveal a species’ origin (e.g. Malayan tapir), habitat (e.g. forest thrush), diet (e.g. giant anteater), and behavior (e.g. splash tetra).

However, there are also signs that identification skills and in-depth knowledge may not be tightly linked. For instance, it has been suggested that children’s ecological knowledge about species continues to rise throughout their primary years while their ability to correctly identify species peaks and then decreases [32]. Moreover, even though people may learn about species from brief exposure via the media or outdoors, such knowledge may remain fragmentary. For instance, a person may encounter a bird in a conifer forest and conclude that the species resides there, without knowing its name, or may recognize an animal that is frequently depicted in cultural sources (e.g. European robin or reindeer on Christmas cards) without knowing its way of life. In line with this, Yli-Panula and Matikainen [30] found that respondents could name native animals, yet they did not link them to the indigenous fen ecosystem where they occurred, and Almeida et al. [33] reported that children placed some well-known animals from the African savannah, zebras and giraffes, in Europe too. If species identification and in-depth species knowledge are not tightly linked, demographic variables such as people’s age and educational level might be more suited for estimating in-depth knowledge, as they are easier to assess and have been reported to correlate with species identification skills [18, 37, 38].

To determine whether identification skills are suitable proxies for in-depth knowledge about species, we explored the two main components of species literacy simultaneously via an online questionnaire distributed among Dutch adults. The questionnaire largely consisted of an animal knowledge test that assessed people’s identification skills and their in-depth knowledge about species’ origin, habitat, diet, and behavior. Subsequently, we compared people’s species identification skills with their in-depth species knowledge, and we determined knowledge gaps and misconceptions. We calculated correlations and odds ratios for in-depth species knowledge and species identification, and we used univariate logistic regression analyses to determine the magnitude of association. As knowledge levels can differ markedly between laypeople and professionals who do work related to biodiversity, and between people of different ages, genders, and educational levels [18], we adjusted for these variables in our exploration of possible associations between the two types of knowledge. Our study provides valuable insights for people who study biodiversity awareness and those who communicate biodiversity, whether in education, research, or conservation, who may wish to use species identification tests in the future to estimate knowledge levels in their target groups.

We investigated the following research questions:

1. How do species identification skills in Dutch adults compare to their level of in-depth knowledge about species per theme (origin, habitat, diet, and behavior) and for themes combined?
2. To what extent does species identification reflect in-depth knowledge about species and how does this compare to alternative predictors (age, gender, educational level, and work)?

## Methods

### Survey design

We designed a questionnaire targeted at Dutch adults, aged 18 years and older (S1 Appendix). The questionnaire consisted largely of an animal knowledge test, presented to participants as an ‘animal quiz’, that covered four themes: origin, habitat, diet, and behavior. To prevent the test from taking too long, each respondent was tested on two randomly selected themes.

Every theme included 15 different vertebrate animal species, making a total of 60: 29 mammals, 24 birds, 3 reptiles, 3 bony fish, and 1 amphibian. Half of the animals were native to the Netherlands, half were exotic. Based on a small pilot study we selected suitable species: we did not include animals with names that would automatically lead respondents to the right answer to the in-depth knowledge question and animals for which multiple answers would be correct (e.g. for theme origin we did not select the Asian elephant or species with a worldwide distribution).

The animals were shown successively, one by one, each represented by one color picture that displayed species-specific morphological characteristics, downloaded from the website https://pixabay.com/. We made sure that pictures did not provide clues to what the correct answer to the in-depth knowledge question might be; if needed we edited the pictures, e.g. by erasing the environmental background. Per animal, two questions were presented: the respondent had to identify the species, and–depending on the theme–answer an in-depth knowledge question about the origin, habitat, diet, or behavior of the species. Both questions were four-answer multiple-choice questions, to avoid difficulties with determining when an answer would be correct; e.g. because of possible spelling mistakes. Careful crafting of the incorrect answer options ensured that respondents would not correctly identify the animal from physical clues in the name (e.g. for the green woodpecker, we included ‘olive woodpecker’ as an incorrect answer).

In addition to the animal quiz, demographic questions were included to assess gender, age (on a 7-point scale), and highest achieved education level (on a 4-point scale). Moreover, we asked participants whether they did voluntary or paid work related to nature, biodiversity, or wild animals; if so, respondents were identified as biodiversity professionals, otherwise as laypeople. The Ethics Review Committee of the Faculty of Science of Leiden University approved this study.

### Data collection and analyses

The questionnaire was constructed in Qualtrics (https://www.qualtrics.com) and distributed online via social media between the 27^th^ of May and 10^th^ of June 2021. After downloading the data from Qualtrics and compiling them in Microsoft Excel 365, we performed descriptive and statistical analysis in IBM SPSS Statistics 25.0.

First, the percentages of correct identifications and correct answers to the in-depth knowledge questions were calculated per theme and in total. In addition, identification rates and in-depth species knowledge rates were calculated per species, to uncover knowledge gaps and misconceptions. Next, we used paired *t*-tests to compare per theme the average levels of the two components of species literacy: species identification and in-depth species knowledge, and we compared the species literacy distributions between laypeople and professionals using Welch’ independent samples *t*-tests.

**Fig 1:**
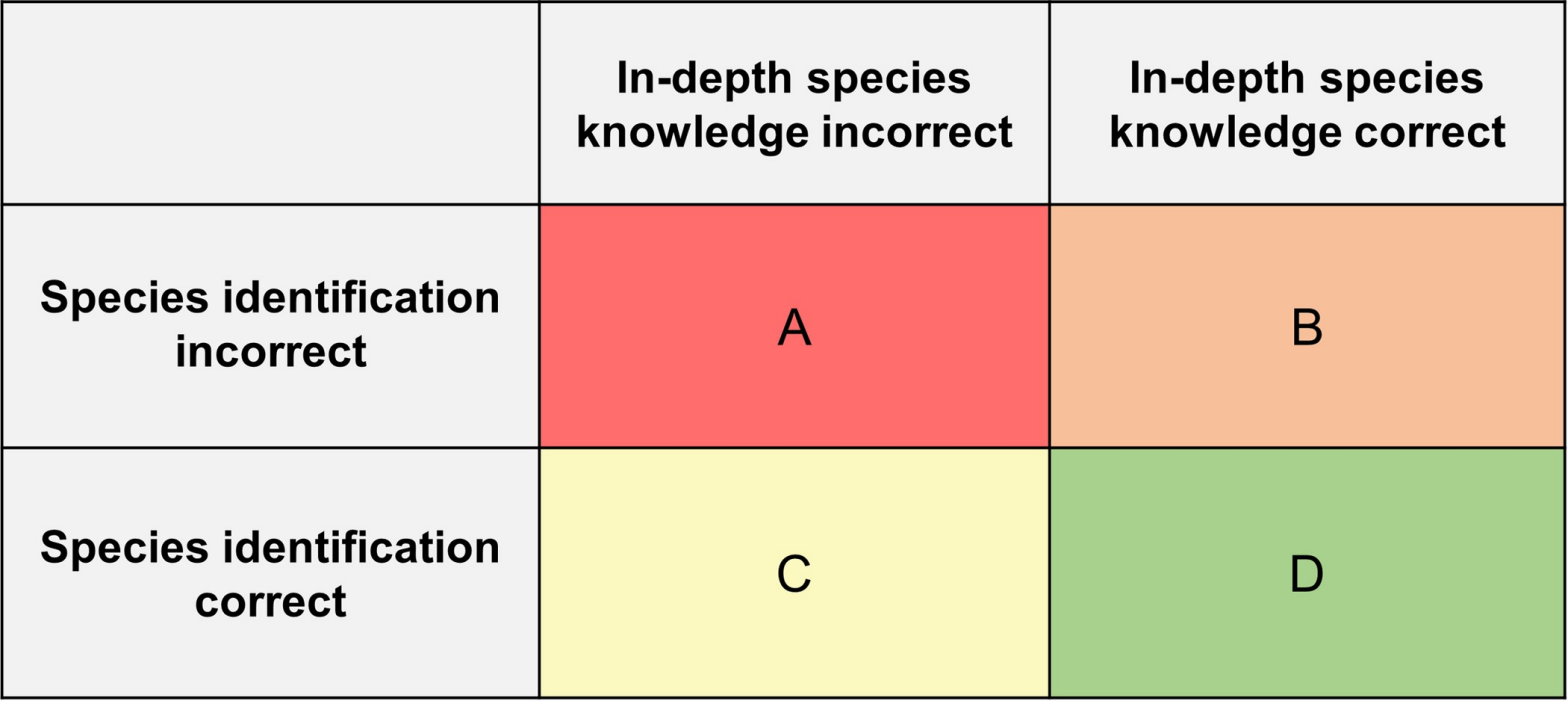
Odds ratios were calculated using the frequency counts in a 2 by 2 contingency table via the following formula: (A*D)/(B*C). Frequency counts of A, B, C, and D were determined per theme and in total.

Subsequently, we investigated the possible association between species identification and in-depth knowledge about species. First, we performed Pearson correlation analyses by assessing the bivariate relationship between species identification and in-depth species knowledge. Then we established the odds ratios (ORs) for in-depth species knowledge among people who did or did not correctly identify species. For this purpose, we determined how frequently both, either, or neither of the identification and corresponding in-depth knowledge question had been answered correctly (Fig 1). We calculated odds ratios with 95% confidence intervals (Cls) for each theme and for all themes combined.

Finally, we conducted univariate regression analysis to determine the extent to which species identification contributed to in-depth knowledge about species, as compared to alternative factors: age, gender, educational level, and work. By including these variables in the model we controlled for biases in the sample of the target group.

## Results

### Descriptive statistics

Of the 8954 respondents who had opened the questionnaire, 1705 were excluded, e.g. because they did not provide consent to participate in the study or because they did not finish the animal knowledge test. The final dataset (S1 Dataset) comprised data from 7249 participants; 1909 indicated that they were professionals (26.3%), and 5259 were identified as laypeople (72.5%). Compared to the 2021 demographic census by Statistics Netherlands (CBS, https://opendata.cbs.nl), the sample was biased towards highly educated citizens (70.8% had achieved higher professional or scientific education against 34.4% of Dutch residents). Moreover, the dataset overrepresented adults under 45 (61.8% against 41.6% of Dutch residents) and women (56.7% against 50.6% of Dutch residents).

### Species literacy levels

On average, participants identified 68.5% of the species correctly. Concerning in-depth knowledge, respondents achieved lower scores (55.0%), particularly for knowledge about species’ diet (49.3%) and behavior (48.8%)–see Table 1. Still, these percentages are considerably higher than the guessing percentage of 25%, indicating that part of the participants knew the correct answers.

**Table 1:** Paired *t*-tests comparing average levels of two components of species literacy: Species identification and in-depth knowledge about species (subdivided into four themes). Each respondent was tested on two themes.

	N	Species identification	In-depth species knowledge	*t*	df	*p*
		(Mean)	(Mean)			
**Origin**	3,494	69.8%	59.7%	41.69	3,493	<0.001
**Habitat**	3,680	69.7%	62.3%	36.66	3,679	<0.001
**Diet**	3,675	67.8%	49.3%	90.10	3,674	<0.001
**Behavior**	3,649	66.6%	48.8%	78.31	3,648	<0.001
**Total**	7,249	68.5%	55.0%	113.51	7,248	<0.001

Knowledge levels were significantly higher in professionals than in laypeople (Table 2). Professionals performed better both at identifying species and at answering in-depth species knowledge questions.

**Table 2:** Welch’ independent samples *t*-tests comparing species literacy levels in laypeople and biodiversity professionals. Two components of species literacy were tested: species identification and in-depth knowledge about species (subdivided into four themes).

	Laypeople		Professionals				
	N	Mean	N	Mean	*t*	df	*p*
**Species identification** **(Total)**	5,259	64.9%	1,909	78.4%	34.59	3,271.39	<0.001
**In-depth species knowledge** **(Total)**	5,259	51.1%	1,909	65.7%	31.64	2,915.39	<0.001
**In-depth species knowledge** **(Origin)**	2,543	55.9%	920	70.0%	18.51	1,527.82	<0.001
**In-depth species knowledge** **(Habitat)**	2,650	58.5%	985	72.2%	22.68	1,590.69	<0.001
**In-depth species knowledge** **(Diet)**	2,681	45.1%	956	61.1%	22.91	1,441.32	<0.001
**In-depth species knowledge** **(Behavior)**	2,644	45.0%	957	59.4%	19.30	1,448.71	<0.001

### Knowledge gaps and misconceptions

Some animals were identified correctly much more frequently than others (S1 Data). For instance, while over 95% of the respondents correctly identified exotic species such as the giant panda, polar bear, and koala, and species native to the Netherlands such as the European mole and robin, less than half of the respondents identified the native grass snake and red-backed shrike, and the exotic leopard seal and black-tailed prairie dog. Hardly anyone correctly identified the gelada, which was often mistaken for the hamadryas baboon even by professionals.

Considering in-depth knowledge, the same pattern was revealed. The origin, habitat, diet, and behavior were shown to be well-known for some species yet largely unknown for others. For example, while most people knew that giant pandas eat bamboo and that white storks make sounds through bill-clattering, a minority of the respondents–including those who correctly identified the animals–knew that black-footed penguins originate from Africa, that okapis reside in rainforests (instead of savannahs), that bearded vultures predominantly eat bones, and that warthogs sleep underground in burrows. Misconceptions about native species were revealed too. Many people were unaware that the European green woodpecker has a diet that mostly consists of ants and instead thought that it mainly eats beetle larvae. Moreover, many respondents wrongly assumed that hares sleep in burrows like rabbits, while they usually do in a shallow depression in the ground, and that shelducks make floating nests, while they usually nest in burrows or cavities.

### Association between in-depth knowledge and species identification

**Fig 2:**
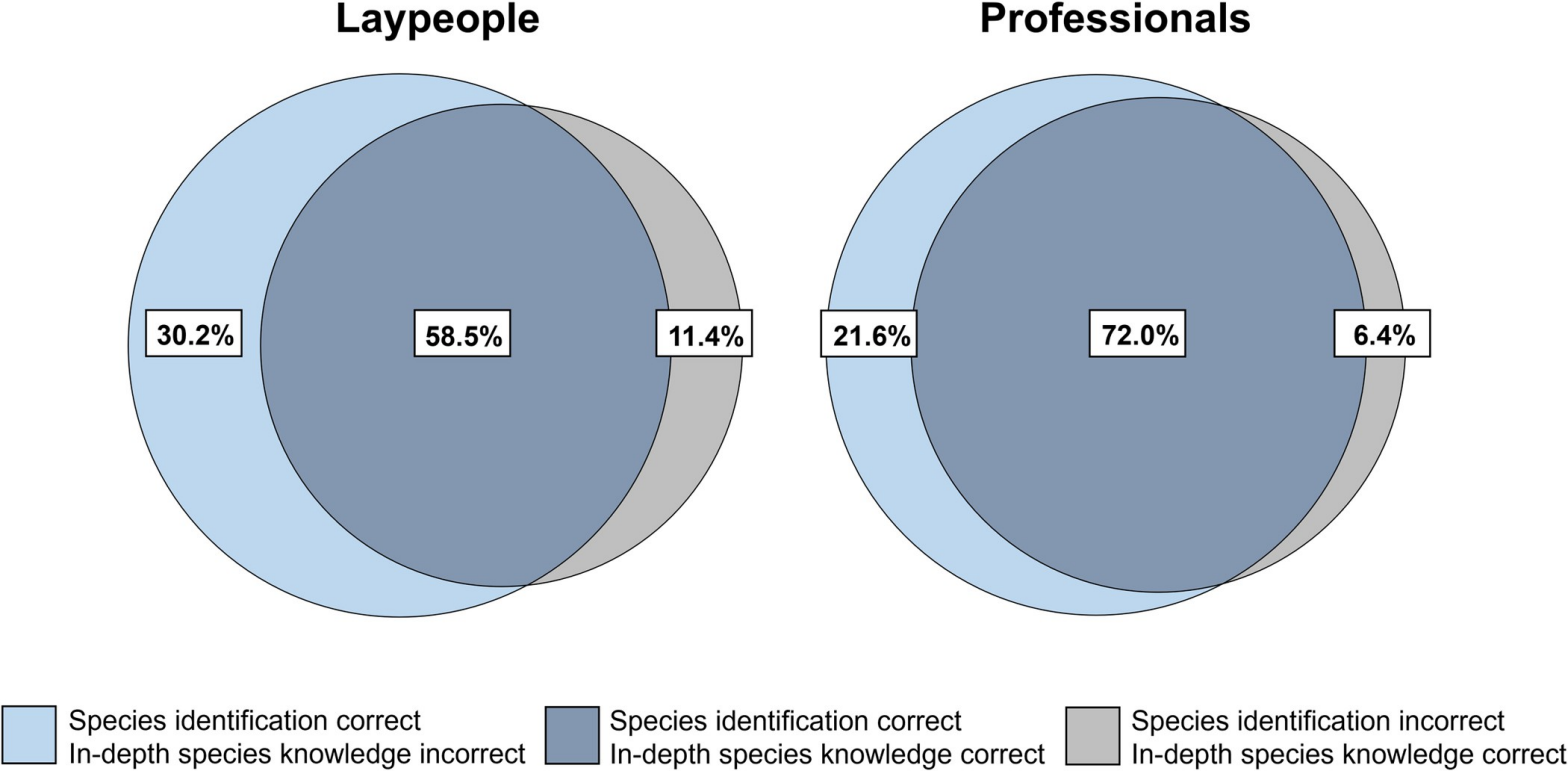
Venn diagrams showing the overlap in species identification and in-depth knowledge in both laypeople and professionals for the four themes combined.

To investigate whether species identification skills are a suitable indicator for in-depth species knowledge, first Venn diagrams were constructed, which showed much overlap between correct species identifications and accurate in-depth species knowledge, especially in professionals (Fig 2). Subsequently, we calculated correlations and odds ratios, and found that in-depth knowledge about species was positively associated with correct identification of those species for the four themes combined (OR: 7.18, 95% CI: 7.04−7.33; *r* = 0.81, *p* < 0.01). In other words, the odds of someone being aware of the origin, habitat, diet, or behavior of an animal were over 7 times larger if the person correctly identified the species. Moreover, an association was found for each theme separately, for knowledge about species’ origin (OR: 5.75, 95% CI: 5.52−5.99; *r* = 0.72, *p* < 0.01), habitat (OR: 5.72, 95% CI: 5.50−5.95; *r* = 0.71, *p* < 0.01), diet (OR: 15.05, 95% CI: 14.31−15.82; *r* = 0.76, *p* < 0.01), and behavior (OR: 6.75, 95% CI: 6.48−7.04; *r* = 0.73, *p* < 0.01), both for professionals and laypeople (S1 File).

As a next step we conducted univariate regression analysis to determine to what extent species identification contributed to in-depth knowledge about species, as compared to alternative factors: age, gender, educational level, and work related to nature, biodiversity, or wild animals (hereafter: ‘work’). Regression models were constructed for each theme of in-depth species knowledge separately and for all themes combined. Species identification was included as a predictor in the model, while age, gender, educational level, and work were added as fixed factors. The assumptions of normally distributed homoscedastic residuals were checked visually; no evidence against these assumptions was found. The percentages reported below are based on the adjusted *R*-squared values.

Species identification and work were significant contributors to the model for each theme and for all themes combined; age, gender and educational level contributed significantly to the models of only some themes (Table 3). Out of all predictor variables, species identification clearly was the most important predictor, explaining in itself 44.2% (origin), 43.5% (habitat), 50,3% (diet), 46.6% (behavior), and 59.7% (themes combined) of the variance in in-depth knowledge about species.

**Table 3:** Regression analyses of predictors of people’s in-depth knowledge about species (subdivided into four themes).

Theme & Variables included in the Model	Type III Sum of Squares	df	Mean Square	*F*	*p*	Partial Eta Squared
**Origin**						
Species identification	11,129.87	1	11,129.87	2,668.64	<0.001	0.442
Work	293.42	1	293.42	70.35	<0.001	0.020
Gender	462.03	1	462.03	110.78	<0.001	0.032
Age	149.14	6	24.86	5.96	<0.001	0.011
Educational level	125.34	3	41.78	10.02	<0.001	0.009
*R*-squared = 0.542 (Adjusted *R*-squared = 0.540)						
**Habitat**						
Species identification	7,718.44	1	7,718.44	2,719.40	<0.001	0.435
Work	306.31	1	306.31	107.92	<0.001	0.030
Gender	283.89	1	283.89	100.02	<0.001	0.028
Age	16.33	6	2.72	0.96	0.452	0.002
Educational level	21.58	3	7.19	2.54	0.055	0.002
*R*-squared = 0.534 (Adjusted *R*-squared = 0.532)						
**Diet**						
Species identification	9,959.56	1	9,959.56	3,586.56	<0.001	0.503
Work	117.47	1	117.47	42.30	<0.001	0.040
Gender	7.64	1	7.64	2.75	0.097	0.037
Age	146.74	6	24.46	8.81	<0.001	0.012
Educational level	13.39	3	4.46	1.61	0.185	0.000
*R*-squared = 0.584 (Adjusted *R*-squared = 0.582)						
**Behavior**						
Species identification	10,624.70	1	10,624.70	3,057.02	<0.001	0.466
Work	418.43	1	418.43	120.40	<0.001	0.033
Gender	99.99	1	99.99	28.77	<0.001	0.008
Age	888.53	6	148.09	42.61	<0.001	0.068
Educational level	18.43	3	6.14	1.77	0.151	0.002
*R*-squared = 0.575 (Adjusted *R*-squared = 0.573)						
**Themes combined**						
Species identification	86,784.91	1	86,784.91	10,331.57	<0.001	0.597
Work	1,445.83	1	1,445.83	172.12	<0.001	0.024
Gender	1,773.53	1	1,773.53	211.14	<0.001	0.029
Age	950.99	6	158.50	18.87	<0.001	0.016
Educational level	163.60	3	54.53	6.49	<0.001	0.003
*R*-squared = 0.681 (Adjusted *R*-squared = 0.680)						

## Discussion

Species identification tests have regularly been used to measure people’s knowledge about species in general, yet without empirical proof that species identification is a good indicator of in-depth knowledge about species. To fill this research gap, we studied the expected link between the two main components of species literacy: species identification skills and in-depth species knowledge, by presenting an animal knowledge test to a large online audience of over 7,000 adult participants.

### Species literacy levels and misconceptions

We found that people were more likely to correctly identify species than to exhibit in-depth knowledge about them. In particular, knowledge about species’ diet and behavior was relatively low. As expected, knowledge levels were significantly higher in professionals than in laypeople. Only a few species, such as the giant panda, polar bear, and robin, were well-known by both professional and lay participants, which links to previous studies that have concluded that people’s perceptions are directed to only a minority of the species that exist [11, 39, 40]. The animals that were identified by most and for which the origin, habitat, diet, or behavior was generally answered correctly can be regarded as charismatic species; they feature frequently in society as cultural representations [41, 42].

We also uncovered misconceptions, some of which seem to stem from generalizations where people extrapolate traits of species’ relatives. For example, many people probably assume incorrectly that all vultures feed on meat from dead animals and that penguins are restricted in range to polar regions. Moreover, we noticed that some animals were frequently confused with a specific other species, which led to in-depth knowledge questions being answered incorrectly; e.g. the jaguar was often misidentified as a leopard and linked to Africa. Similarly, while virtually all respondents who recognized the cuckoo knew that the bird lays her eggs in the nest of another bird, those who misidentified the bird hardly ever chose the correct answer.

Misconceptions and misidentifications can have serious implications, e.g. when venomous and nonvenomous species are confused. In our study, people who misidentified the native nonvenomous grass snake as an adder or as a black mamba usually assumed that the snake was venomous, which links to Corbett et al. [43], who reported that participants tended to believe that many of the nonvenomous snakes presented to them were venomous. From a conservation perspective, this is unfortunate, as species that are deemed to be a risk to people’s health may experience persecution. Furthermore, as laypeople were unaware of the way of life of certain animals, notably common, native species such as hares, green woodpeckers, and shelducks, they miss out on opportunities to enrich their lives, e.g. by growing a sense of place [44]. The results demonstrate that there is plenty of room for educators to broaden people’s perceptions.

### Association between species identification and in-depth knowledge

As noted above, people were more likely to correctly identify species than to exhibit in-depth knowledge about them. For a considerable number of species (e.g. warthog, common eider, coconut lorikeet), only a minority of respondents who correctly identified them answered the in-depth knowledge question correctly, in line with studies that reported a lack of deeper understanding about animals that could be named [30, 33]. This could be an indication that often people become familiar with the name or physical characteristics of an animal first, enabling them to accurately identify it, after which in-depth knowledge may or may not follow. Furthermore, people may learn isolated facts about species from brief exposure (e.g. via the media) and this knowledge may remain fragmentary, which may also explain that species identification did not mirror in-depth species knowledge perfectly.

Still, identification skills do not have to be perfect reflections of in-depth knowledge about species in order to serve as proxies. Thus, using correlation and regression analyses, we investigated to what extent species identification skills reflect in-depth knowledge about species. The odds for having in-depth knowledge about species were considerably higher for those who correctly identified species as compared with those who did not correctly identify species, both for knowledge about species’ origin, habitat, diet, and behavior. Moreover, species identification was by far the best predictor for in-depth species knowledge in comparison to other factors (work, age, gender, and educational level). Although our respondents were all from the Netherlands, we have no reason to doubt that our results have international applicability, as species identification tests have revealed similar knowledge patterns in different countries.

## Conclusion

In conclusion, we provide evidence that species identification skills are associated with in-depth knowledge about species. Species identification can predict in-depth species knowledge reasonably well, and a lot better than demographic characteristics such as age and highest achieved educational level, which underscores the value of using species identification tests to assess what people know about animals. However, as people tended to experience more difficulty with the in-depth knowledge questions than with the identification of the species, and as misconceptions were uncovered about species that were correctly identified, researchers and communicators should take into account that such tests hold limitations. Such restrictions may depend on the animal group that is included in a test and the type of in-depth knowledge that is assessed, something which future research could elucidate. Moreover, future studies could determine whether the association between identification and in-depth knowledge also applies to taxa such as plants and fungi.

Communicators could use a variety of short quizzes to address different knowledge components in their target audiences. A mix of such assessments could help them in becoming aware of current knowledge levels and existing misconceptions. By adjusting their communication accordingly, they will be able to engage the public more effectively on the topic of biodiversity. Moreover, we recommend educators who aim to expand species literacy in their audiences to embed species in context, e.g. by sharing information about how they can be identified and combining this with fun facts and background information about their living environment, diet, or behavior. This can connect people with the vast diversity of life that exists worldwide and in the local environment, which can ultimately help build broad-based public support for conservation.

## Supporting information

S1 Appendix Questionnaire. (PDF)

S1 Dataset Final dataset. (XLSX)

S1 Data Knowledge levels. (XLSX)

S1 File Odds ratios (ORs) for in-depth species knowledge among professionals and laypeople who did or did not correctly identify species, and Pearson correlations between species identification and in-depth species knowledge (subdivided into four themes). (DOCX)

## References

[1] Thompson RA, Zamboanga BL. Prior knowledge and its relevance to student achievement in introduction to psychology. Teach Psychol. 2003;30(2):96–101.

[2] Buijs AE, Fischer A, Rink D, Young JC. Looking beyond superficial knowledge gaps: Understanding public representations of biodiversity. Int J Biodivers Sci Manag. 2008;4(2):65–80.

[3] Hailikari T, Katajavuori N, Lindblom-Ylänne S. The relevance of prior knowledge in learning and instructional design. Am J Pharm Educ. 2008;72(5):1–8. 10.5688/aj7205113 PMC2630138 19214267

[4] Mortimer JAJ, Greene TC, Mortimer SP. Assessing bird vocalisation identification accuracy using a computer-based quiz. New Zeal J Zool. 2019;46(3):201–24.

[5] Perrenet JC. Levels of thinking in computer science: Development in bachelor students’ conceptualization of algorithm. Educ Inf Technol. 2010;15(2):87–107.

[6] Dickens C, Lambert BL, Cromwell T, Piano MR. Nurse overestimation of patients’ health literacy. J Health Commun. 2013;18(sup1):62–9. doi: 10.1080/10810730.2013.825670 24093346 PMC3814908

[7] Kelly PA, Haidet P. Physician overestimation of patient literacy: A potential source of health care disparities. Patient Educ Couns. 2007;66(1):119–22. doi: 10.1016/j.pec.2006.10.007 17140758

[8] Hooykaas MJD, Aten C, Hemelaar EM, Albers CJ, Schilthuizen M, Smeets I. Children’s species literacy as estimated and desired by biodiversity communicators: A mismatch with the actual level. bioRxiv. 2021;1–15.

[9] Burgman MA. Trusting judgements: How to get the best out of experts. 1st ed. Cambridge, UK: Cambridge University Press; 2016. 214 p.

[10] Tetlock PE, Gardner D. Supervoorspellers: Goed voorspellen is een manier van denken–en iedereen kan het leren. (Superforecasting). Amsterdam/Antwerpen: Business Contact; 2015. 318 p.

[11] Ballouard JM, Brischoux F, Bonnet X. Children prioritize virtual exotic biodiversity over local biodiversity. PLoS One. 2011;6(8). doi: 10.1371/journal.pone.0023152 21829710 PMC3150400

[12] Mohneke M, Erguvan F, Schlüter K. Explorative study about knowledge of species in the field of early years education. J Emergent Sci. 2016;(11):11–22.

[13] Nyhus PJ, Sumianto, Tilson R. Wildlife knowledge among migrants in southern Sumatra, Indonesia: Implications for conservation. Environ Conserv. 2003;30(2):192–9.

[14] Randler C. Pupils’ factual knowledge about vertebrate species. J Balt Sci Educ [Internet]. 2008;7(1):48–54. Available from: http://oaji.net/articles/2014/987-1404289078.pdf

[15] Randler C, Wieland L. Knowledge about common vertebrate species in German kindergarten pupils. J Balt Sci Educ [Internet]. 2010;9(2):135–41. Available from: http://oaji.net/articles/2014/987-1405171515.pdf

[16] Vázquez-Plass E., Wunderle JM. Differences in knowledge about birds and their conservation between rural and urban residents of Puerto Rico. J Caribb Ornithol [Internet]. 2010;23(2):93–100. Available from: https://www.jco.birdscaribbean.org/index.php/jco/article/view/334

[17] Gerl T, Randler C, Neuhaus BJ. Vertebrate species knowledge: an important skill is threatened by extinction. Int J Sci Educ. 2021;43(6):1–21.

[18] Hooykaas MJD, Schilthuizen M, Aten C, Hemelaar EM, Albers CJ, Smeets I. Identification skills in biodiversity professionals and laypeople: A gap in species literacy. Biol Conserv. 2019;238:108202.

[19] Remmele M, Lindemann-Matthies P. Like father, like son? On the relationship between parents’ and children’s familiarity with species and sources of knowledge about plants and animals. Eurasia J Math Sci Technol Educ. 2018;14(10):em1581.

[20] Dixon SP, Birchenough A, Evans SM, Quigley MP. Children’s knowledge of birds: How can it be improved and can it be used to conserve wildlife? Trans Nat Hist Soc Northumbria. 2005;64:121–34.

[21] Peterson MN, Sternberg M, Lopez A, Liu J. Ocelot awareness among Latinos on the Texas and Tamaulipas border. Hum Dimens Wildl An Int J. 2008;13(5):339–47.

[22] Pilgrim SE, Smith DJ, Pretty J. A cross-regional assessment of the factors affecting ecoliteracy: Implications for policy and practice. Ecol Appl. 2007;17(6):1742–51. doi: 10.1890/06-1358.1 17913137

[23] Kai Z, Woan TS, Jie L, Goodale E, Kitajima K, Bagchi R, et al. Shifting baselines on a tropical forest frontier: Extirpations drive declines in local ecological knowledge. PLoS One. 2014;9(1):e86598. doi: 10.1371/journal.pone.0086598 24466163 PMC3897741

[24] Balmford A, Clegg L, Coulson T, Taylor J. Why conservationists should heed Pokémon. Science (80-). 2002;295(5564):2367b. doi: 10.1126/science.295.5564.2367b 11924673

[25] Elder J, Coffin C, Farrior M. Engaging the public on biodiversity. Madison, United States; 1998.

[26] Novacek MJ. Engaging the public in technology policy. PNAS. 2008;105(Suppl. 1):11571–8. 18695244 10.1073/pnas.0802599105 PMC2556408

[27] Wilson C, Tisdell C. Knowledge of birds and willingness to support their conservation: An Australian case study. Bird Conserv Int. 2005;15(3):225–35.

[28] Greene HW. Organisms in nature as a central focus for biology. Trends Ecol Evol. 2005;20(1):23–7. doi: 10.1016/j.tree.2004.11.005 16701336

[29] Kubiatko M, Prokop P. Pupils’ misconceptions about mammals. J Balt Sci Educ. 2007;6(1):5–15.

[30] Yli-Panula E, Matikainen E. Students and student teachers’ ability to name animals in ecosystems: A perspective of animal knowledge and biodiversity. J Balt Sci Educ. 2014;13(4):559–72.

[31] Torkar G. Young Slovenian learners’ knowledge about animal diversity on different continents. Int J Biol Educ. 2016;5(1):1–11.

[32] Huxham M, Welsh A, Berry A, Templeton S. Factors influencing primary school children’s knowledge of wildlife. J Biol Educ. 2006;41(1):9–12.

[33] Almeida A, García Fernández B, Strecht-Ribeiro O. Children’s knowledge and contact with native fauna: A comparative study between Portugal and Spain. J Biol Educ. 2020;54(1):17–32.

[34] Barker S, Slingsby D. From nature table to niche: curriculum progression in ecological concepts. Int J Sci Educ. 1998;20(4):479–86.

[35] Leather SR, Quicke DJL. Where would Darwin have been without taxonomy? J Biol Educ. 2009;43(2):51–2.

[36] Tunnicliffe SD, Reiss MJ. Building a model of the environment: How do children see animals? J Biol Educ. 1999;33(3):142–8.

[37] Randler C. Animal related activities as determinants of species knowledge. Eurasia J Math Sci Technol Educ. 2010;6(4):237–43.

[38] Randler C, Höllwarth A, Schaal S. Urban park visitors and their knowledge of animal species. Anthrozoos. 2007;20(1):65–74.

[39] Lindemann‐Matthies P. ‘Loveable’ mammals and ‘lifeless’ plants: How children’s interest in common local organisms can be enhanced through observation of nature. Int J Sci Educ. 2005;27(6):655–77.

[40] Davies T, Cowley A, Bennie J, Leyshon C, Inger R, Carter H, et al. Popular interest in vertebrates does not reflect extinction risk and is associated with bias in conservation investment. PLoS One. 2018;13(9):e0203694. doi: 10.1371/journal.pone.0203694 30256838 PMC6157853

[41] Albert C, Luque GM, Courchamp F. The twenty most charismatic species. PLoS One. 2018;13(7):e0199149. doi: 10.1371/journal.pone.0199149 29985962 PMC6037359

[42] Courchamp F, Jaric I, Albert C, Yves M, Ripple WJ, Chapron G. The paradoxical extinction of the most charismatic animals. PLoS Biol. 2018;16(4, e2003997). doi: 10.1371/journal.pbio.2003997 29649205 PMC5896884

[43] Corbett SW, Anderson B, Nelson B, Bush S, Hayes WK, Cardwell MD. Most lay people can correctly identify indigenous venomous snakes. Am J Emerg Med. 2005;23(6):759–62. doi: 10.1016/j.ajem.2005.03.008 16182984

[44] Horwitz P, Lindsay M, O’Connor M. Biodiversity, endemism, sense of place, and public health: Inter-relationships for Australian Inland aquatic systems. Ecosyst Heal. 2001;7(4):253–65.

