# Supplementary material for: Species identification skills predict in-depth knowledge about species
Source: PLoS One. 2022 Apr 18;17(4):e0266972. doi: 10.1371/journal.pone.0266972 (PMC9015127; doi:10.1371/journal.pone.0266972)
Supplement: S1 Appendix — (PDF) [file pone.0266972.s001.pdf]

Questionnaire

This document contains the questionnaire used for the research project. The questionnaire was distributed in Dutch, so both the original Dutch version and an English translation have been included.

Note: In this document the names of researchers, institute and department have been removed for the Double Blind Peer Review process.

Questionnaire (English Translation) 2

Answer Sheets Animal Quiz (English Translation) 4

Theme Origin: Where do animals come from? 4

Theme Habitat: In which habitat does this animal usually occur? 6

Theme Diet: What does this animal usually eat? 8

Theme Behavior: How does this animal naturally behave? 10

Questionnaire (Original Dutch Version) 12

Antwoordbladen Dieren Quiz (Original Dutch Version) 14

Thema Herkomst: Waar komt dit dier van nature voor? 14

Thema Leefomgeving: In welke omgeving leeft dit dier gewoonlijk? 16

Thema Voedsel: Wat eet dit dier gewoonlijk? 18

Thema Gedrag: Hoe gedraagt dit dier zich van nature? 20

Pictures used in the Animal Knowledge Test 22

Origin 22

Habitat 23

Diet 25

Behavior 27

Questionnaire (English Translation)

Introduction
How skilled are you at identifying animal species? And what do you know about animals? By participating in our animal quiz you help us with our research on animal knowledge of the Dutch public (18 years and older). The quiz takes 10 to 12 minutes to complete and your answers will be processed and stored anonymously by the department NAME INSTITUTE AND DEPARTEMENT.

Thank you very much for your participation! NAMES/CONTACT DETAILS

Consent
By clicking "Agree", you consent to the provision of some personal data, which will be processed anonymously. The data resulting from this questionnaire will be stored in accordance with the General Data Protection Regulation (GDPR) of the European Union.
o Agree

Demographic Intro
We would like to reach a diverse audience with our study, so we will first ask you a few questions about your background.

Age
What is your age in years?
o 18 - 24
o 25 - 34
o 35 - 44
o 45 - 54
o 55 - 64
o 65 - 74
o 75 years or older
o I'd rather not say

Gender
How do you identify yourself?
o Man
o Woman
o Different
o I'd rather not say

Education Level
What is your highest achieved level of education?
o Primary education
o Lower secondary education VMBO, HAVO, or VWO, or MBO 1 (or something similar)
o Upper secondary education HAVO or VWO, or MBO 2-4 (or something similar)
o HBO or WO (or something similar)

Work
Do you do (voluntary) work that has to do with nature, biodiversity and/or wild animals?
o Yes
o No
o I don’t know

Hobby
Do you have a hobby that has to do with nature, biodiversity and/or wild animals? (e.g. bird watching, keeping tropical fish, or watching nature films)
o Yes
o No
o I don’t know

Start Quiz
The animal quiz includes 2 rounds with 15 animals each! You will see the animals one by one.

Please note: in each round there are animals that few people know - so the quiz is a challenge! If you don't know an answer, take a guess. We take this into account in our research. It is important for our study that you do not look up the answers and ask no one else for help. In addition, do not take the animal quiz more than once.

Have fun and good luck!

Hereafter two out of four themes are randomly selected by Qualtrics and presented as two quiz rounds to the respondent; below are the introductory sentences per theme. Every theme includes 15 animals that will be presented to the respondent one by one. For each species two four answer multiple choice questions are asked.

Theme Origin: *Round "Where do animals come from?"*
Do you know the following animals and do you know from which continent they come? Whether you take a guess or not, thank you for participating!
(This theme includes 15 animal species. For each animal first the respondent is asked 'What is the name of this animal species?', followed by a second question: Where does this animal occur naturally?)

Theme Habitat: *Round "Where do animals live?"*
Do you know the following animals and in what environment they usually occur? Whether or not you know the answers, thank you for participating!
(This theme includes 15 animal species. For each animal first the respondent is asked 'What is the name of this animal species?', followed by a second question: In what environment does this animal usually occur?)

Theme Diet: *Round “What do animals eat?”*
Do you know the following animals and what they usually eat? Whether you have to take a guess or not, we really appreciate you taking part.
(This theme includes 15 animal species. For each animal first the respondent is asked 'What is the name of this animal species?', followed by a second question: What does this animal usually eat?)

Theme Behavior: *Round "Behavior!"*
Do you know the following animals and how they behave naturally? No matter how difficult or easy you find the questions, you will help us enormously by doing this quiz!
(This theme includes 15 animal species. For each animal first the respondent is asked 'What is the name of this animal species?', followed by a second question: How does this animal naturally behave? This question was specified per species)

Answer Sheets Animal Quiz (English Translation)

Theme Origin: Where do animals come from?<br>Species Identification								
**O_nr.**	**Scientific Name**	**Class**	**Type**	**Question**	**Correct Answer**	**Incorrect answer 1**	**Incorrect answer 2**	**Incorrect answer 3**
1	*Bison bison*	Mammal	Exotic	What is the name of this animal species?	American bison	Gaur	Wild yak	African buffalo
2	*Paracheirodon axelrodi*	Bony fish	Exotic	What is the name of this animal species?	Cardinal tetra	Cherry barb	Neon damselfish	Rainbow krib
3	*Panthera onca*	Mammal	Exotic	What is the name of this animal species?	Jaguar	Marbled cat	Leopard	Cheetah
4	*Hylobates lar*	Mammal	Exotic	What is the name of this animal species?	White-handed gibbon	Squirrel monkey	Bonobo	Indri
5	*Salamandra salamandra*	Amphibian	Native	What is the name of this animal species?	Fire salamander	Tokeh	Common leopard gecko	Tiger salamander
6	*Alligator mississippiensis*	Reptile	Exotic	What is the name of this animal species?	American alligator	Gharial	Black caiman	Nile crocodile
7	*Meles meles*	Mammal	Native	What is the name of this animal species?	European badger	Raccoon	Opossum	Skunk
8	*Pyrrhula pyrrhula*	Bird	Native	What is the name of this animal species?	Eurasian bullfinch	Asian rosy finch	Pine siskin	Masked lovebird
9	*Dromaius novaehollandiae*	Bird	Exotic	What is the name of this animal species?	Emu	Moa	Greater rhea	Ostrich
10	*Hydrochoerus hydrochaeris*	Mammal	Exotic	What is the name of this animal species?	Capybara	Muskrat	Greater glider	Pika
11	*Spheniscus demersus*	Bird	Exotic	What is the name of this animal species?	Black-footed penguin	Adélie penguin	Marbled murrelet	Pigeon guillemot
12	*Vombatus ursinus*	Mammal	Exotic	What is the name of this animal species?	Common wombat	Hoary marmot	Degu	Common hamster
13	*Sagittarius serpentarius*	Bird	Exotic	What is the name of this animal species?	Secretarybird	Demoiselle crane	Cassowary	Crested seriema
14	*Puma concolor*	Mammal	Exotic	What is the name of this animal species?	Cougar	Clouded leopard	Iberian lynx	Golden cat
15	*Pygocentrus nattereri*	Bony fish	Exotic	What is the name of this animal species?	Red-bellied piranha	Spotfin lionfish	Jewel cichlid	Firemouth cichlid

Theme Origin: Where do animals come from?<br>In-depth Knowledge About Species								
O_nr.	Scientific Name	Class	Type	Question	Correct answer	Incorrect answer 1	Incorrect answer 2	Incorrect answer 3
1	*Bison bison*	Mammal	Exotic	Where does this animal occur naturally?	North America	Asia	South America	Africa
2	*Paracheirodon axelrodi*	Bony fish	Exotic	Where does this animal occur naturally?	South America	Asia	Australia	Africa
3	*Panthera onca*	Mammal	Exotic	Where does this animal occur naturally?	South America	Asia	Australia	Africa
4	*Hylobates lar*	Mammal	Exotic	Where does this animal occur naturally?	Asia	South America	Australia	Africa
5	*Salamandra salamandra*	Amphibian	Native	Where does this animal occur naturally?	Europe	Asia	Australia	North America
6	*Alligator mississippiensis*	Reptile	Exotic	Where does this animal occur naturally?	North America	Asia	South America	Africa
7	*Meles meles*	Mammal	Native	Where does this animal occur naturally?	Europe	South America	Australia	North America
8	*Pyrrhula pyrrhula*	Bird	Native	Where does this animal occur naturally?	Europe	Australia	North America	Africa
9	*Dromaius novaehollandiae*	Bird	Exotic	Where does this animal occur naturally?	Australia	Asia	South America	Africa
10	*Hydrochoerus hydrochaeris*	Mammal	Exotic	Where does this animal occur naturally?	South America	Europe	Australia	North America
11	*Spheniscus demersus*	Bird	Exotic	Where does this animal occur naturally?	Africa	Europe	Antarctica	North America
12	*Vombatus ursinus*	Mammal	Exotic	Where does this animal occur naturally?	Australia	Europe	South America	North America
13	*Sagittarius serpentarius*	Bird	Exotic	Where does this animal occur naturally?	Africa	Asia	Australia	South America
14	*Puma concolor*	Mammal	Exotic	Where does this animal occur naturally?	North America	Asia	Europe	Africa
15	*Pygocentrus nattereri*	Bony fish	Exotic	Where does this animal occur naturally?	South America	Australia	Africa	North America

Theme Habitat: In which habitat does this animal usually occur?<br>Species Identification								
**H_nr.**	**Scientific Name**	**Class**	**Type**	**Question**	**Correct Answer**	**Incorrect answer 1**	**Incorrect answer 2**	**Incorrect answer 3**
1	*Suricata suricatta*	Mammal	Exotic	What is the name of this animal species?	Meerkat	Pine marten	Least weasel	Javan mongoose
2	*Rupicapra rupicapra*	Mammal	Exotic	What is the name of this animal species?	Chamois	Mouflon	Oribi	Springbok
3	*Regulus regulus*	Bird	Native	What is the name of this animal species?	Goldcrest	Eurasian wren	Western yellow wagtail	Red-breasted flycatcher
4	*Okapia johnstoni*	Mammal	Exotic	What is the name of this animal species?	Okapi	Quagga	Pronghorn	Oryx
5	*Dama dama*	Mammal	Native	What is the name of this animal species?	Fallow deer	Reindeer	Sitka deer	Water deer
6	*Loxia curvirostra*	Bird	Native	What is the name of this animal species?	Common crossbill	Common scissorsbill	Common crossfinch	Common scissorsfinch
7	*Cynomys ludovicianus*	Mammal	Exotic	What is the name of this animal species?	Black-tailed prairie dog	Alpine marmot	Wood lemming	Common gundi
8	*Sciurus vulgaris*	Mammal	Native	What is the name of this animal species?	Eurasian red squirrel	Souslik	Sugar glider	Harris's antelope squirrel
9	*Panurus biarmicus*	Bird	Native	What is the name of this animal species?	Bearded reedling	Ortolan	Oxpecker	Splendid fairywren
10	*Heterocephalus glaber*	Mammal	Exotic	What is the name of this animal species?	Naked mole-rat	Naked marsupial shrew	Naked ground squirrel	Naked gopher
11	*Bucorvus leadbeateri*	Bird	Exotic	What is the name of this animal species?	Southern ground hornbill	Great frigatebird	Black hornbill	Thick-billed raven
12	*Tapirus indicus*	Mammal	Exotic	What is the name of this animal species?	Malayan tapir	Bolivian moussafa	Tamandua	Giant anteater
13	*Limosa limosa*	Birds	Native	What is the name of this animal species?	Black-tailed godwit	Eurasian woodcock	Black-winged stilt	Ruddy turnstone
14	*Vulpes zerda*	Mammal	Exotic	What is the name of this animal species?	Fennec fox	Arctic fox	Golden jackal	Serval
15	*Ursus maritimus*	Mammal	Exotic	What is the name of this animal species?	Polar bear	Kodiak bear	Grizzly bear	Sloth bear

Theme Habitat: In which habitat does this animal usually occur?<br>In-depth Knowledge About Species								
**H_nr.**	**Scientific Name**	**Class**	**Type**	**Question**	**Correct answer**	**Incorrect answer 1**	**Incorrect answer 2**	**Incorrect answer 3**
1	*Suricata suricatta*	Mammal	Exotic	In what environment does this animal usually occur?	Savanna	Marsh	Mixed forest (Deciduous and conifer trees)	Rainforest
2	*Rupicapra rupicapra*	Mammal	Exotic	In what environment does this animal usually occur?	High mountainous environment	Desert	Polar region	Savanna
3	*Regulus regulus*	Bird	Native	In what environment does this animal usually occur?	Conifer forest	Mangrove forest	Reed beds	Grasssland
4	*Okapia johnstoni*	Mammal	Exotic	In what environment does this animal usually occur?	Rainforest	Conifer forest	Savanna	Desert
5	*Dama dama*	Mammal	Native	In what environment does this animal usually occur?	Deciduous forest	Polar region	Conifer forest	Marsh
6	*Loxia curvirostra*	Bird	Native	In what environment does this animal usually occur?	Conifer forest	Mangrove forest	Grasssland	Reed beds
7	*Cynomys ludovicianus*	Mammal	Exotic	In what environment does this animal usually occur?	Grasssland	High mountainous environment	Polar region	Mixed forest (Deciduous and conifer trees)
8	*Sciurus vulgaris*	Mammal	Native	In what environment does this animal usually occur?	Mixed forest (Deciduous and conifer trees)	Grasssland	Rainforest	Desert
9	*Panurus biarmicus*	Bird	Native	In what environment does this animal usually occur?	Reedbeds	Deciduous forest	Savanna	Mangrove forest
10	*Heterocephalus glaber*	Mammal	Exotic	In what environment does this animal usually occur?	Desert	Rainforest	Marsh	Conifer forest
11	*Bucorvus leadbeateri*	Bird	Exotic	In what environment does this animal usually occur?	Savanna	Mangrove forest	Rainforest	High mountainous environment
12	*Tapirus indicus*	Mammal	Exotic	In what environment does this animal usually occur?	Rainforest	Savanna	Conifer forest	Grasssland
13	*Limosa limosa*	Birds	Native	In what environment does this animal usually occur?	Grasssland	Deciduous forest	Conifer forest	High mountainous environment
14	*Vulpes zerda*	Mammal	Exotic	In what environment does this animal usually occur?	Desert	Polar region	Mixed forest (Deciduous and conifer trees)	Reed beds
15	*Ursus maritimus*	Mammal	Exotic	In what environment does this animal usually occur?	Polar region	Deciduous forest	Marsh	Rainforest

Theme Diet: What does this animal usually eat?

Species Identification

D_nr.	Scientific Name	Class	Type	Question	Correct Answer	Incorrect answer 1	Incorrect answer 2	Incorrect answer 3
1	*Alcedo atthis*	Bird	Native	What is the name of this animal species?	Common kingfisher	Emerald hummingbird	Lesser spotted woodpecker	White-throated bee-eater
2	*Carduelis carduelis*	Bird	Native	What is the name of this animal species?	European goldfinch	Bohemian waxwing	Whinchat	European pied flycatcher
3	*Lutra lutra*	Mammal	Native	What is the name of this animal species?	Eurasian otter	European polecat	Stone marten	Beaver
4	*Falco peregrinus*	Bird	Native	What is the name of this animal species?	Peregrine falcon	Common kestrel	Common buzzard	European honey buzzard
5	*Picus viridis*	Bird	Native	What is the name of this animal species?	European green woodpecker	Olive woodpecker	Red-crowned woodpecker	Ivory-billed woodpecker
6	*Talpa europaea*	Mammal	Native	What is the name of this animal species?	European mole	Burying beetle	Tundra vole	Pygmy shrew
7	*Gypaetus barbatus*	Bird	Exotic	What is the name of this animal species?	Bearded vulture	King vulture	Golden eagle	Andean condor
8	*Hirundo rustica*	Bird	Native	What is the name of this animal species?	Barn swallow	Blue-backed manakin	Fork-tailed woodnymph	Paradise whydah
9	*Somateria mollissima*	Bird	Native	What is the name of this animal species?	Common eider	Common merganser	Smew	Common loon
10	*Phascolarctos cinereus*	Mammal	Exotic	What is the name of this animal species?	Koala	Lesser panda	Gray woolly monkey	Aye-aye
11	*Trichoglossus haematodus*	Bird	Exotic	What is the name of this animal species?	Coconut lorikeet	Australian king parrot	Eastern rosella	Imperial amazon
12	*Amblyrhynchus cristatus*	Reptile	Exotic	What is the name of this animal species?	Marine iguana	Fiji crested iguana	Gila monster	Common chuckwalla
13	*Canis lupus*	Mammal	Native	What is the name of this animal species?	Wolf	Black-backed jackal	Maned wolf	Raccoon dog
14	*Theropithecus gelada*	Mammal	Exotic	What is the name of this animal species?	Gelada	Hamadryas baboon	Mandril	Northern pig-tailed macaque
15	*Ailuropoda melanoleuca*	Mammal	Exotic	What is the name of this animal species?	Giant panda	Spectacled bear	Sun bear	Asian black bear

Theme Diet: What does this animal usually eat?<br>In-depth Knowledge About Species								
**D_nr.**	**Scientific Name**	**Class**	**Type**	**Question**	**Correct answer**	**Incorrect answer 1**	**Incorrect answer 2**	**Incorrect answer 3**
1	*Alcedo atthis*	Bird	Native	What does this animal usually eat?	Fish	Nectar	Nuts	Insects
2	*Carduelis carduelis*	Bird	Native	What does this animal usually eat?	Seeds	Fruits	Insects	Nectar
3	*Lutra lutra*	Mammal	Native	What does this animal usually eat?	Fish	Eggs	Rodents	Plants
4	*Falco peregrinus*	Bird	Native	What does this animal usually eat?	Birds	Rodents	Carrion (meat of dead animals)	Insects
5	*Picus viridis*	Bird	Native	What does this animal usually eat?	Ants	Beetle larvae	Earthworms	Nuts
6	*Talpa europaea*	Mammal	Native	What does this animal usually eat?	Earthworms	Carrion (meat of dead animals)	Tuberous roots	Ants
7	*Gypaetus barbatus*	Bird	Exotic	What does this animal usually eat?	Bones	Eggs	Rodents	Carrion (meat of dead animals)
8	*Hirundo rustica*	Bird	Native	What does this animal usually eat?	Insects	Fruits	Nectar	Seeds
9	*Somateria mollissima*	Bird	Native	What does this animal usually eat?	Shellfish	Grass	Seaweed	Fish
10	*Phascolarctos cinereus*	Mammal	Exotic	What does this animal usually eat?	Tree leaves	Bamboo shoots	Fruits	Beetle larvae
11	*Trichoglossus haematodus*	Bird	Exotic	What does this animal usually eat?	Nectar	Nuts	Insects	Seeds
12	*Amblyrhynchus cristatus*	Reptile	Exotic	What does this animal usually eat?	Seaweed	Grass	Insects	Eggs
13	*Canis lupus*	Mammal	Native	What does this animal usually eat?	Ungulates	Carrion (meat of dead animals)	Birds	Rodents
14	*Theropithecus gelada*	Mammal	Exotic	What does this animal usually eat?	Grass	Fruits	Tree leaves	Nuts
15	*Ailuropoda melanoleuca*	Mammal	Exotic	What does this animal usually eat?	Bamboo shoots	Tree leaves	Fruits	Tuberous roots

Theme Behavior: How does this animal naturally behave? Species Identification								
**B_nr.**	**Scientific Name**	**Class**	**Type**	**Question**	**Correct Answer**	**Incorrect answer 1**	**Incorrect answer 2**	**Incorrect answer 3**
1	*Ciconia ciconia*	Bird	Native	What is the name of this animal species?	White stork	Cattle egret	Milky stork	Common crane
2	*Capreolus capreolus*	Mammal	Native	What is the name of this animal species?	Roe deer	Muntjac	Musk deer	Indian hog deer
3	*Cervus elaphus*	Mammal	Native	What is the name of this animal species?	Red deer	Mule deer	Chital	Moose
4	*Erithacus rubecula*	Bird	Native	What is the name of this animal species?	European robin	Cirl bunting	Brambling	Chaffinch
5	*Hydrurga leptonyx*	Mammal	Exotic	What is the name of this animal species?	Leopard seal	Steller sea lion	Weddell seal	Monk seal
6	*Aegithalos caudatus*	Bird	Native	What is the name of this animal species?	Long-tailed tit	Penduline tit	Marsh tit	Willow tit
7	*Cuculus canorus*	Bird	Native	What is the name of this animal species?	Common cuckoo	Sparrowhawk	Nightjar	Mauritius fody
8	*Tadorna tadorna*	Bird	Native	What is the name of this animal species?	Common shelduck	Harlequin duck	Common pochard	Northern shoveler
9	*Lycaon pictus*	Mammal	Exotic	What is the name of this animal species?	African wild dog	Hyena	Bat-eared fox	Coyote
10	*Esox lucius*	Bony fish	Native	What is the name of this animal species?	Northern pike	Perch	Asp	Garfish
11	*Natrix natrix*	Reptile	Native	What is the name of this animal species?	Grass snake	Common adder	Calabar python	Black mamba
12	*Aythya fuligula*	Bird	Native	What is the name of this animal species?	Tufted duck	Whiteflankduck	Common teal	Gadwall
13	*Lanius collurio*	Bird	Native	What is the name of this animal species?	Red-backed shrike	Chestnut-backed chickadee	Short-toed treecreeper	Rock nuthatch
14	*Lepus europaeus*	Mammal	Native	What is the name of this animal species?	European hare	European rabbit	Pika	Marsh rabbit
15	*Phacochoerus africanus*	Mammal	Exotic	What is the name of this animal species?	Common warthog	Bearded pig	Wild boar	Bushpig

Theme Behavior: How does this animal naturally behave?<br>In-depth Knowledge About Species								
**B_nr.**	**Scientific Name**	**Class**	**Type**	**Question**	**Correct answer**	**Incorrect answer 1**	**Incorrect answer 2**	**Incorrect answer 3**
1	*Ciconia ciconia*	Bird	Native	What sound is characteristic of this animal?	Clattering with the bill	A deep "boom" from the pharynx/throat	Singing of the wings during flight	Ticking of the legs
2	*Capreolus capreolus*	Mammal	Native	What sound is characteristic of this animal?	Barking	Cooing	Bleating	Oinking
3	*Cervus elaphus*	Mammal	Native	What sound is characteristic of this animal?	Rutting	Snorting	Bubbling	Braying
4	*Erithacus rubecula*	Bird	Native	How does this animal live outside the breeding season?	Solitary (alone)	Pairwise (Male + female)	Group of males ór females	Mixed group of females and males
5	*Hydrurga leptonyx*	Mammal	Exotic	How does this animal live outside the breeding season?	Solitary (alone)	Pairwise (Male + female)	Group of males ór females	Mixed group of females and males
6	*Aegithalos caudatus*	Bird	Native	How does this animal live outside the breeding season?	Mixed group of females and males	Solitary (alone)	Pairwise (Male + female)	Group of males ór females
7	*Cuculus canorus*	Bird	Native	Where does this animal usually lay its eggs?	In the nest of a different species	On top of a rock or cliff	High up in a tree	In a hanging nest
8	*Tadorna tadorna*	Bird	Native	Where does this animal usually lay its eggs?	In burrows or cavities	In a floating nest	Underwater	At a clearing in the sand or grass
9	*Lycaon pictus*	Mammal	Exotic	How does this animal usually get its food?	By chasing prey for a long time	By stalking and surprising prey	By stealing prey from another animal	By eating leftovers of prey from another animal
10	*Esox lucius*	Bony fish	Native	How does this animal usually get its food?	By stalking and surprising prey	By chasing prey for a long time	By stealing prey from another animal	By eating leftovers of prey from another animal
11	*Natrix natrix*	Reptile	Native	How does this animal usually kill its prey?	By swallowing it whole	By strangling it	By killing it with its fangs that channel venom	By chewing it to pieces
12	*Aythya fuligula*	Bird	Native	How does this animal usually look for food?	He dives underwater	He goes ashore	He searches for food at the water surface	It flies with an open beak
13	*Lanius collurio*	Bird	Native	How does this animal usually store its food?	Impaled in a bush	In a nest	Buried in the ground	Between tree bark
14	*Lepus europaeus*	Mammal	Native	Where does this animal usually sleep?	In a shallow depression in the ground	Underground in a burrow	At a spot of high altitude	In high grass
15	*Phacochoerus africanus*	Mammal	Exotic	Where does this animal usually sleep?	Underground in a burrow	In a shallow depression in the ground	At a spot of high altitude	In high grass

Questionnaire (Original Dutch Version)

Introductie
Hoe goed kunt u diersoorten herkennen? En wat weet u over dieren? Door mee te doen met onze dierenquiz helpt u ons met ons onderzoek naar dierenkennis van het volwassen Nederlands publiek (18 jaar en ouder). De quiz duurt 10 tot 12 minuten en uw antwoorden worden anoniem verwerkt en opgeslagen door de afdeling NAAM INSTITUUT EN AFDELING.

Hartelijk bedankt voor uw deelname! NAMEN/CONTACTGEGEVENS

Consent
Door op "Akkoord" te klikken, gaat u akkoord met het verstrekken van enkele persoonlijke gegevens, die volledig anoniem zullen worden verwerkt. De gegevens die volgen uit deze enquête worden bewaard in overeenstemming met de Algemene Verordening Gegevensbescherming (AVG) van de Europese Unie.
o Akkoord

Demografie
We willen met ons onderzoek graag een divers publiek bereiken en stellen u daarom eerst een aantal vragen over uw achtergrond.

Leeftijd
Wat is uw leeftijd in jaren?
o 18 - 24
o 25 - 34
o 35 - 44
o 45 - 54
o 55 - 64
o 65 - 74
o 75 jaar of ouder
o Zeg ik liever niet

Gender
Hoe identificeert u zichzelf?
o Man
o Vrouw
o Anders
o Zeg ik liever niet

Opleidingsniveau
Wat is uw hoogst behaalde opleiding?
o Basisonderwijs
o Onderbouw vmbo, havo, of vwo, of MBO 1 (of iets vergelijkbaars)
o Bovenbouw havo of vwo, of MBO 2-4 (of iets vergelijkbaars)
o HBO of WO (of iets vergelijkbaars)

Werk
Doet u (vrijwilligers)werk dat te maken heeft met natuur, biodiversiteit en/of wilde dieren?
o Ja
o Nee
o Weet ik niet

Hobby
Heeft u een hobby die te maken heeft met natuur, biodiversiteit en/of wilde dieren? (bijv. vogels kijken, aquariumvissen houden, of natuurfilms kijken)
o Ja
o Nee
o Weet ik niet

Start Quiz
De dierenquiz bestaat uit 2 rondes met ieder 15 dieren! U krijgt de dieren één voor één te zien.

Let op: in elke ronde zitten dieren die weinig mensen kennen - de quiz is dus een uitdaging! Weet u een antwoord niet, doe dan een gok. We houden hiermee rekening in ons onderzoek. Voor onze studie is het belangrijk dat u de antwoorden niet opzoekt en niemand anders om hulp vraagt. Doe de dierenquiz bovendien niet meer dan één keer.

Veel plezier, en succes!

Hierna worden random twee thema's gekozen en voorgelegd als twee quizronden aan de respondent. Hieronder staan de introducties van ieder thema.

Thema Herkomst: *Ronde "Waar komen dieren vandaan?"*
Weet u van de volgende dieren welke het zijn en uit welk werelddeel ze komen? Of u moet gokken of niet, bedankt voor uw deelname!
(Dit thema bevat 15 diersoorten – zie overzicht. Voor elk dier wordt eerst gevraagd 'Wat is de naam van deze diersoort?', waarna de volgende vraag volgt: Waar komt dit dier van nature voor?)

Thema Leefomgeving: *Ronde "Waar leven dieren?"*
Weet u van de volgende dieren welke het zijn en in welke omgeving ze gewoonlijk leven? Of u de antwoorden wel of niet weet, bedankt dat u meedoet!
(Dit thema bevat 15 diersoorten – zie overzicht. Voor elk dier wordt eerst gevraagd 'Wat is de naam van deze diersoort?', waarna de volgende vraag volgt: In welke omgeving leeft dit dier gewoonlijk?)

Thema Voedsel: *Ronde "Voedsel!"*
Weet u van de volgende dieren welke het zijn en wat ze van nature eten? Of u moet gokken of niet, we waarderen het zeer dat u meedoet.
(Dit thema bevat 15 diersoorten – zie overzicht. Voor elk dier wordt eerst gevraagd 'Wat is de naam van deze diersoort?', waarna de volgende vraag volgt: What does this animal usually eat?)

Thema Gedrag: *Ronde "Gedrag!"*
Weet u van de volgende dieren welke het zijn en hoe ze zich van nature gedragen? Hoe moeilijk of makkelijk u de vragen ook vindt, u helpt ons enorm door mee te doen aan deze quiz!
(Dit thema bevat 15 diersoorten – zie overzicht. Voor elk dier wordt eerst gevraagd 'Wat is de naam van deze diersoort?', waarna de volgende vraag volgt: Hoe gedraagt dit dier zich van nature? Deze vraag was gespecificeerd per diersoort)

Antwoordbladen Dieren Quiz (Original Dutch Version)

Thema Herkomst: Waar komt dit dier van nature voor?<br>Soortidentificatie								
**O_nr.**	**Wetenschappelijke naam**	**Klasse**	**Type**	**Vraag**	**Juist Antwoord**	**Onjuist Antwoord 1**	**Onjuist Antwoord 2**	**Onjuist Antwoord 3**
1	*Bison bison*	Zoogdier	Exotisch	Wat is de naam van deze diersoort?	Bizon	Gaur	Jak	Kafferbuffel
2	*Paracheirodon axelrodi*	Beenvis	Exotisch	Wat is de naam van deze diersoort?	Kardinaaltetra	Sherrybarbeel	Neonjuffer	Kersenbuikcichlide
3	*Panthera onca*	Zoogdier	Exotisch	Wat is de naam van deze diersoort?	Jaguar	Marmerkat	Luipaard	Cheetah
4	*Hylobates lar*	Zoogdier	Exotisch	Wat is de naam van deze diersoort?	Withandgibbon	Doodshoofdaapje	Bonobo	Indri
5	*Salamandra salamandra*	Amfibie	Inheems	Wat is de naam van deze diersoort?	Vuursalamander	Tokeh	Luipaardgekko	Tijgersalamander
6	*Alligator mississippiensis*	Reptiel	Exotisch	Wat is de naam van deze diersoort?	Mississippialligator	Gaviaal	Zwarte kaaiman	Nijlkrokodil
7	*Meles meles*	Zoogdier	Inheems	Wat is de naam van deze diersoort?	Das	Wasbeer	Opossum	Stinkdier
8	*Pyrrhula pyrrhula*	Vogel	Inheems	Wat is de naam van deze diersoort?	Goudvink	Roze bergvink	Dennensijs	Masker-agapornis
9	*Dromaius novaehollandiae*	Vogel	Exotisch	Wat is de naam van deze diersoort?	Emoe	Moa	Nandoe	Struisvogel
10	*Hydrochoerus hydrochaeris*	Zoogdier	Exotisch	Wat is de naam van deze diersoort?	Capibara	Muskusrat	Reuzenkoeskoes	Pika
11	*Spheniscus demersus*	Vogel	Exotisch	Wat is de naam van deze diersoort?	Zwartvoetpinguïn	Adeliepinguïn	Marmeralk	Duifzeekoet
12	*Vombatus ursinus*	Zoogdier	Exotisch	Wat is de naam van deze diersoort?	Wombat	Grijze marmot	Degoe	Korenwolf
13	*Sagittarius serpentarius*	Vogel	Exotisch	Wat is de naam van deze diersoort?	Secretarisvogel	Jufferkraan	Kasuaris	Kuifseriema
14	*Puma concolor*	Zoogdier	Exotisch	Wat is de naam van deze diersoort?	Poema	Nevelpanter	Pardellynx	Goudkat
15	*Pygocentrus nattereri*	Beenvis	Exotisch	Wat is de naam van deze diersoort?	Roodbuikpiranha	Rode koraalduivel	Rode acara	Vuurkeelcichlide

Thema Herkomst: Waar komt dit dier van nature voor?<br>Dieptekennis Over Soorten								
**O_nr.**	**Wetenschappelijke naam**	**Klasse**	**Type**	**Vraag**	**Juist antwoord**	**Onjuist Antwoord 1**	**Onjuist Antwoord 2**	**Onjuist Antwoord 3**
1	*Bison bison*	Zoogdier	Exotisch	Waar komt dit dier van nature voor?	Noord-Amerika	Azië	Zuid-Amerika	Afrika
2	*Paracheirodon axelrodi*	Beenvis	Exotisch	Waar komt dit dier van nature voor?	Zuid-Amerika	Azië	Australië	Afrika
3	*Panthera onca*	Zoogdier	Exotisch	Waar komt dit dier van nature voor?	Zuid-Amerika	Azië	Australië	Afrika
4	*Hylobates lar*	Zoogdier	Exotisch	Waar komt dit dier van nature voor?	Azië	Zuid-Amerika	Australië	Afrika
5	*Salamandra salamandra*	Amfibie	Inheems	Waar komt dit dier van nature voor?	Europa	Azië	Australië	Noord-Amerika
6	*Alligator mississippiensis*	Reptiel	Exotisch	Waar komt dit dier van nature voor?	Noord-Amerika	Azië	Zuid-Amerika	Afrika
7	*Meles meles*	Zoogdier	Inheems	Waar komt dit dier van nature voor?	Europa	Zuid-Amerika	Australië	Noord-Amerika
8	*Pyrrhula pyrrhula*	Vogel	Inheems	Waar komt dit dier van nature voor?	Europa	Australië	Noord-Amerika	Afrika
9	*Dromaius novaehollandiae*	Vogel	Exotisch	Waar komt dit dier van nature voor?	Australië	Azië	Zuid-Amerika	Afrika
10	*Hydrochoerus hydrochaeris*	Zoogdier	Exotisch	Waar komt dit dier van nature voor?	Zuid-Amerika	Europa	Australië	Noord-Amerika
11	*Spheniscus demersus*	Vogel	Exotisch	Waar komt dit dier van nature voor?	Afrika	Europa	Antarctica	Noord-Amerika
12	*Vombatus ursinus*	Zoogdier	Exotisch	Waar komt dit dier van nature voor?	Australië	Europa	Zuid-Amerika	Noord-Amerika
13	*Sagittarius serpentarius*	Vogel	Exotisch	Waar komt dit dier van nature voor?	Afrika	Azië	Australië	Zuid-Amerika
14	*Puma concolor*	Zoogdier	Exotisch	Waar komt dit dier van nature voor?	Noord-Amerika	Azië	Europa	Afrika
15	*Pygocentrus nattereri*	Beenvis	Exotisch	Waar komt dit dier van nature voor?	Zuid-Amerika	Australië	Afrika	Noord-Amerika

Thema Leefomgeving: In welke omgeving leeft dit dier gewoonlijk?<br>Soortidentificatie								
H_nr.	Wetenschappelijke naam	Klasse	Type	Vraag	Juist Antwoord	Onjuist Antwoord 1	Onjuist Antwoord 2	Onjuist Antwoord 3
1	*Suricata suricatta*	Zoogdier	Exotisch	Wat is de naam van deze diersoort?	Stokstaartje	Boommarter	Wezel	Indische mangoeste
2	*Rupicapra rupicapra*	Zoogdier	Exotisch	Wat is de naam van deze diersoort?	Gems	Moeflon	Oribi	Springbok
3	*Regulus regulus*	Vogel	Inheems	Wat is de naam van deze diersoort?	Goudhaan	Winterkoning	Gele kwikstaart	Kleine vliegenvanger
4	*Okapia johnstoni*	Zoogdier	Exotisch	Wat is de naam van deze diersoort?	Okapi	Quagga	Pronghorn	Oryx
5	*Dama dama*	Zoogdier	Inheems	Wat is de naam van deze diersoort?	Damhert	Rendier	Sitkahert	Waterree
6	*Loxia curvirostra*	Vogel	Inheems	Wat is de naam van deze diersoort?	Kruisbek	Schaarbek	Kruisvink	Schaarvink
7	*Cynomys ludovicianus*	Zoogdier	Exotisch	Wat is de naam van deze diersoort?	Zwartstaartprairiehond	Alpenmarmot	Boslemming	Noord-Afrikaanse goendi
8	*Sciurus vulgaris*	Zoogdier	Inheems	Wat is de naam van deze diersoort?	Rode eekhoorn	Soeslik	Suikereekhoorn	Harrisgrondeekhoorn
9	*Panurus biarmicus*	Vogel	Inheems	Wat is de naam van deze diersoort?	Baardman	Ortolaan	Ossenpikker	Prachtelfje
10	*Heterocephalus glaber*	Zoogdier	Exotisch	Wat is de naam van deze diersoort?	Naakte molrat	Naakte buidelmuis	Naakte grondeekhoorn	Naakte goffer
11	*Bucorvus leadbeateri*	Vogel	Exotisch	Wat is de naam van deze diersoort?	Zuidelijke hoornraaf	Grote fregatvogel	Zwarte jaarvogel	Dikbekraaf
12	*Tapirus indicus*	Zoogdier	Exotisch	Wat is de naam van deze diersoort?	Maleise tapir	Boliviaanse moussafa	Tamandoea	Reuzenmiereneter
13	*Limosa limosa*	Vogels	Inheems	Wat is de naam van deze diersoort?	Grutto	Houtsnip	Steltkluut	Steenloper
14	*Vulpes zerda*	Zoogdier	Exotisch	Wat is de naam van deze diersoort?	Fennek	Poolvos	Goudjakhals	Serval
15	*Ursus maritimus*	Zoogdier	Exotisch	Wat is de naam van deze diersoort?	IJsbeer	Kodiakbeer	Grizzlybeer	Lippenbeer

Thema Leefomgeving: In welke omgeving leeft dit dier gewoonlijk?<br>Dieptekennis Over Soorten								
**H_nr.**	**Wetenschappelijke naam**	**Klasse**	**Type**	**Vraag**	**Juist antwoord**	**Onjuist Antwoord 1**	**Onjuist Antwoord 2**	**Onjuist Antwoord 3**
1	*Suricata suricatta*	Zoogdier	Exotisch	In welke omgeving leeft dit dier gewoonlijk?	Savanne	Moeras	Gemengd bos (loof- en naaldbomen)	Regenwoud
2	*Rupicapra rupicapra*	Zoogdier	Exotisch	In welke omgeving leeft dit dier gewoonlijk?	Hooggebergte	Woestijn	Poolgebied	Savanne
3	*Regulus regulus*	Vogel	Inheems	In welke omgeving leeft dit dier gewoonlijk?	Naaldbos	Mangrovebos	Rietland	Grasland
4	*Okapia johnstoni*	Zoogdier	Exotisch	In welke omgeving leeft dit dier gewoonlijk?	Regenwoud	Naaldbos	Savanne	Woestijn
5	*Dama dama*	Zoogdier	Inheems	In welke omgeving leeft dit dier gewoonlijk?	Loofbos	Poolgebied	Naaldbos	Moeras
6	*Loxia curvirostra*	Vogel	Inheems	In welke omgeving leeft dit dier gewoonlijk?	Naaldbos	Mangrovebos	Grasland	Rietland
7	*Cynomys ludovicianus*	Zoogdier	Exotisch	In welke omgeving leeft dit dier gewoonlijk?	Grasland	Hooggebergte	Poolgebied	Gemengd bos (loof- en naaldbomen)
8	*Sciurus vulgaris*	Zoogdier	Inheems	In welke omgeving leeft dit dier gewoonlijk?	Gemengd bos (loof- en naaldbomen)	Grasland	Regenwoud	Woestijn
9	*Panurus biarmicus*	Vogel	Inheems	In welke omgeving leeft dit dier gewoonlijk?	Rietland	Loofbos	Savanne	Mangrovebos
10	*Heterocephalus glaber*	Zoogdier	Exotisch	In welke omgeving leeft dit dier gewoonlijk?	Woestijn	Regenwoud	Moeras	Naaldbos
11	*Bucorvus leadbeateri*	Vogel	Exotisch	In welke omgeving leeft dit dier gewoonlijk?	Savanne	Mangrovebos	Regenwoud	Hooggebergte
12	*Tapirus indicus*	Zoogdier	Exotisch	In welke omgeving leeft dit dier gewoonlijk?	Regenwoud	Savanne	Naaldbos	Grasland
13	*Limosa limosa*	Vogels	Inheems	In welke omgeving leeft dit dier gewoonlijk?	Grasland	Loofbos	Naaldbos	Hooggebergte
14	*Vulpes zerda*	Zoogdier	Exotisch	In welke omgeving leeft dit dier gewoonlijk?	Woestijn	Poolgebied	Gemengd bos (loof- en naaldbomen)	Rietland
15	*Ursus maritimus*	Zoogdier	Exotisch	In welke omgeving leeft dit dier gewoonlijk?	Poolgebied	Loofbos	Moeras	Regenwoud

Thema Voedsel: Wat eet dit dier gewoonlijk?
Soortidentificatie

D_nr.	Wetenschappelijke naam	Klasse	Type	Vraag	Juist Antwoord	Onjuist Antwoord 1	Onjuist Antwoord 2	Onjuist Antwoord 3
1	*Alcedo atthis*	Vogel	Inheems	Wat is de naam van deze diersoort?	IJsvogel	Smaragdkolibri	Kleine bonte specht	Witkeelbijeneter
2	*Carduelis carduelis*	Vogel	Inheems	Wat is de naam van deze diersoort?	Putter	Pestvogel	Paapje	Bonte vliegenvanger
3	*Lutra lutra*	Zoogdier	Inheems	Wat is de naam van deze diersoort?	Otter	Bunzing	Steenmarter	Bever
4	*Falco peregrinus*	Vogel	Inheems	Wat is de naam van deze diersoort?	Slechtvalk	Torenvalk	Buizerd	Wespendief
5	*Picus viridis*	Vogel	Inheems	Wat is de naam van deze diersoort?	Groene specht	Olijfspecht	Roodkruinspecht	Ivoorsnavelspecht
6	*Talpa europaea*	Zoogdier	Inheems	Wat is de naam van deze diersoort?	Mol	Doodgraver	Noordse woelmuis	Dwergspitsmuis
7	*Gypaetus barbatus*	Vogel	Exotisch	Wat is de naam van deze diersoort?	Lammergier	Koningsgier	Steenarend	Andescondor
8	*Hirundo rustica*	Vogel	Inheems	Wat is de naam van deze diersoort?	Boerenzwaluw	Prachtmanakin	Vorkstaartbosnimf	Paradijswida
9	*Somateria mollissima*	Vogel	Inheems	Wat is de naam van deze diersoort?	Eidereend	Grote zaagbek	Nonnetje	IJsduiker
10	*Phascolarctos cinereus*	Zoogdier	Exotisch	Wat is de naam van deze diersoort?	Koala	Kleine panda	Grijze wolaap	Aye aye
11	*Trichoglossus haematodus*	Vogel	Exotisch	Wat is de naam van deze diersoort?	Regenbooglori	Australische koningsparkiet	Prachtrosella	Keizeramazone
12	*Amblyrhynchus cristatus*	Reptiel	Exotisch	Wat is de naam van deze diersoort?	Zeeleguaan	Gekamde fijileguaan	Gilamonster	Chuckwalla
13	*Canis lupus*	Zoogdier	Inheems	Wat is de naam van deze diersoort?	Wolf	Zadeljakhals	Manenwolf	Wasbeerhond
14	*Theropithecus gelada*	Zoogdier	Exotisch	Wat is de naam van deze diersoort?	Gelada	Mantelbaviaan	Mandril	Leeuwmakaak
15	*Ailuropoda melanoleuca*	Zoogdier	Exotisch	Wat is de naam van deze diersoort?	Reuzenpanda	Brilbeer	Maleise beer	Kraagbeer

Thema Voedsel: Wat eet dit dier gewoonlijk?<br>Dieptekennis Over Soorten								
**D_nr.**	**Wetenschappelijke naam**	**Klasse**	**Type**	**Vraag**	**Juist antwoord**	**Onjuist Antwoord 1**	**Onjuist Antwoord 2**	**Onjuist Antwoord 3**
1	*Alcedo atthis*	Vogel	Inheems	Wat eet dit dier gewoonlijk?	Vissen	Nectar	Noten	Insecten
2	*Carduelis carduelis*	Vogel	Inheems	Wat eet dit dier gewoonlijk?	Zaden	Vruchten	Insecten	Nectar
3	*Lutra lutra*	Zoogdier	Inheems	Wat eet dit dier gewoonlijk?	Vissen	Eieren	Knaagdieren	Planten
4	*Falco peregrinus*	Vogel	Inheems	Wat eet dit dier gewoonlijk?	Vogels	Knaagdieren	Aas (vlees van dode dieren)	Insecten
5	*Picus viridis*	Vogel	Inheems	Wat eet dit dier gewoonlijk?	Mieren	Keverlarven	Regenwormen	Noten
6	*Talpa europaea*	Zoogdier	Inheems	Wat eet dit dier gewoonlijk?	Regenwormen	Aas (vlees van dode dieren)	Wortelknollen	Mieren
7	*Gypaetus barbatus*	Vogel	Exotisch	Wat eet dit dier gewoonlijk?	Botten	Eieren	Knaagdieren	Aas (vlees van dode dieren)
8	*Hirundo rustica*	Vogel	Inheems	Wat eet dit dier gewoonlijk?	Insecten	Vruchten	Nectar	Zaden
9	*Somateria mollissima*	Vogel	Inheems	Wat eet dit dier gewoonlijk?	Schelpdieren	Gras	Zeewier	Vissen
10	*Phascolarctos cinereus*	Zoogdier	Exotisch	Wat eet dit dier gewoonlijk?	Boombladeren	Bamboescheuten	Vruchten	Keverlarven
11	*Trichoglossus haematodus*	Vogel	Exotisch	Wat eet dit dier gewoonlijk?	Nectar	Noten	Insecten	Zaden
12	*Amblyrhynchus cristatus*	Reptiel	Exotisch	Wat eet dit dier gewoonlijk?	Zeewier	Gras	Insecten	Eieren
13	*Canis lupus*	Zoogdier	Inheems	Wat eet dit dier gewoonlijk?	Hoefdieren	Aas (vlees van dode dieren)	Vogels	Knaagdieren
14	*Theropithecus gelada*	Zoogdier	Exotisch	Wat eet dit dier gewoonlijk?	Gras	Vruchten	Boombladeren	Noten
15	*Ailuropoda melanoleuca*	Zoogdier	Exotisch	Wat eet dit dier gewoonlijk?	Bamboescheuten	Boombladeren	Vruchten	Wortelknollen

Thema Gedrag: Hoe gedraagt dit dier zich van nature?
Soortidentificatie

B_nr.	Wetenschappelijke naam	Klasse	Type	Vraag	Juist Antwoord	Onjuist Antwoord 1	Onjuist Antwoord 2	Onjuist Antwoord 3
1	*Ciconia ciconia*	Vogel	Inheems	Wat is de naam van deze diersoort?	Ooievaar	Koereiger	Nimmerzat	Kraanvogel
2	*Capreolus capreolus*	Zoogdier	Inheems	Wat is de naam van deze diersoort?	Ree	Muntjak	Muskushert	Zwijnshert
3	*Cervus elaphus*	Zoogdier	Inheems	Wat is de naam van deze diersoort?	Edelhert	Muildierhert	Axishert	Eland
4	*Erithacus rubecula*	Vogel	Inheems	Wat is de naam van deze diersoort?	Roodborst	Cirlgors	Keep	Vink
5	*Hydrurga leptonyx*	Zoogdier	Exotisch	Wat is de naam van deze diersoort?	Zeeluipaard	Stellerzeeleeuw	Weddellzeehond	Monniksrob
6	*Aegithalos caudatus*	Vogel	Inheems	Wat is de naam van deze diersoort?	Staartmees	Buidelmees	Glanskop	Matkop
7	*Cuculus canorus*	Vogel	Inheems	Wat is de naam van deze diersoort?	Koekoek	Sperwer	Nachtzwaluw	Mauritiuswever
8	*Tadorna tadorna*	Vogel	Inheems	Wat is de naam van deze diersoort?	Bergeend	Harlekijneend	Tafeleend	Slobeend
9	*Lycaon pictus*	Zoogdier	Exotisch	Wat is de naam van deze diersoort?	Afrikaanse wilde hond	Hyena	Lepelhond	Coyote
10	*Esox lucius*	Beenvis	Inheems	Wat is de naam van deze diersoort?	Snoek	Baars	Roofblei	Geep
11	*Natrix natrix*	Reptiel	Inheems	Wat is de naam van deze diersoort?	Ringslang	Adder	Aardpython	Zwarte mamba
12	*Aythya fuligula*	Vogel	Inheems	Wat is de naam van deze diersoort?	Kuifeend	Witflankeend	Wintertaling	Krakeend
13	*Lanius collurio*	Vogel	Inheems	Wat is de naam van deze diersoort?	Grauwe klauwier	Kastanjerugmees	Boomkruiper	Rotsklever
14	*Lepus europaeus*	Zoogdier	Inheems	Wat is de naam van deze diersoort?	Europese haas	Europees konijn	Fluithaas	Moeraskonijn
15	*Phacochoerus africanus*	Zoogdier	Exotisch	Wat is de naam van deze diersoort?	Wrattenzwijn	Baardzwijn	Everzwijn	Penseelzwijn

Thema Gedrag: Hoe gedraagt dit dier zich van nature?<br>Dieptekennis Over Soorten								
**B_nr.**	**Wetenschappelijke naam**	**Klasse**	**Type**	**Vraag**	**Juist antwoord**	**Onjuist Antwoord 1**	**Onjuist Antwoord 2**	**Onjuist Antwoord 3**
1	*Ciconia ciconia*	Vogel	Inheems	Welk geluid is kenmerkend voor dit dier?	Geklepper met de snavel	Een lage "hoemp" uit de keelholte	Zingen van de vleugels tijdens het vliegen	Getik van de poten
2	*Capreolus capreolus*	Zoogdier	Inheems	Welk geluid is kenmerkend voor dit dier?	Blaffen	Kirren	Mekkeren	Knorren
3	*Cervus elaphus*	Zoogdier	Inheems	Welk geluid is kenmerkend voor dit dier?	Burlen	Briesen	Balderen	Balken
4	*Erithacus rubecula*	Vogel	Inheems	Hoe leeft dit dier buiten het broedseizoen?	Solitair (alleen)	Paarsgewijs (mannetje + vrouwtje)	Groep van mannetjes óf vrouwtjes	Gemengde groep van vrouwtjes en mannetjes
5	*Hydrurga leptonyx*	Zoogdier	Exotisch	Hoe leeft dit dier buiten het broedseizoen?	Solitair (alleen)	Paarsgewijs (mannetje + vrouwtje)	Groep van mannetjes óf vrouwtjes	Gemengde groep van vrouwtjes en mannetjes
6	*Aegithalos caudatus*	Vogel	Inheems	Hoe leeft dit dier buiten het broedseizoen?	Gemengde groep van vrouwtjes en mannetjes	Solitair (alleen)	Paarsgewijs (mannetje + vrouwtje)	Groep van mannetjes óf vrouwtjes
7	*Cuculus canorus*	Vogel	Inheems	Waar legt dit dier gewoonlijk haar eieren?	In het nest van een andere soort	Op een rots of klif	Hoog in een boom	In een hangend nest
8	*Tadorna tadorna*	Vogel	Inheems	Waar legt dit dier gewoonlijk haar eieren?	In holen of holtes	In een drijvend nest	Onder water	Op een open plek in het zand of gras
9	*Lycaon pictus*	Zoogdier	Exotisch	Hoe komt dit dier gewoonlijk aan zijn voedsel?	Door een prooi langdurig te achtervolgen	Door een prooi te besluipen en te verrassen	Door een verse prooi af te pakken van een ander dier	Door de restjes op te eten van de prooi van een ander dier
10	*Esox lucius*	Beenvis	Inheems	Hoe komt dit dier gewoonlijk aan zijn voedsel?	Door een prooi te besluipen en te verrassen	Door een prooi langdurig te achtervolgen	Door een verse prooi af te pakken van een ander dier	Door de restjes op te eten van de prooi van een ander dier
11	*Natrix natrix*	Reptiel	Inheems	Hoe doodt dit dier gewoonlijk zijn prooi?	Door hem in zijn geheel in te slikken	Door hem te wurgen	Door hem te doden met zijn giftanden	Door hem kapot te kauwen
12	*Aythya fuligula*	Vogel	Inheems	Hoe zoekt dit dier gewoonlijk zijn voedsel?	Hij duikt onder water	Hij gaat aan land	Hij zoekt aan het wateroppervlak	Hij vliegt met open snavel
13	*Lanius collurio*	Vogel	Inheems	Hoe bewaart dit dier gewoonlijk zijn voedsel?	Opgeprikt in een struik	In een nest	Ingegraven in de grond	Tussen boomschors
14	*Lepus europaeus*	Zoogdier	Inheems	Waar slaapt dit dier gewoonlijk?	In een ondiepe kuil	Ondergronds in een hol	Op een hooggelegen plek	In hoog gras
15	*Phacochoerus africanus*	Zoogdier	Exotisch	Waar slaapt dit dier gewoonlijk?	Ondergronds in een hol	In een ondiepe kuil	Op een hooggelegen plek	In hoog gras

Pictures used in the Animal Knowledge Test

All pictures were freely downloaded from the website https://pixabay.com/ (Pixabay License), https://freepngimg.com/ or from Wikimedia commons.

Origin

1	Species: American bison - *Bison bison*<br>Source:<br>https://pixabay.com/nl/photos/bison-bull-herbivoren-geile-vee-4665984/<br><br>Pixabay-user: AlLes (Alexander Lesnitsky)
2	Species: Cardinal tetra - *Paracheirodon axelrodi*<br>Source:<br>https://pixabay.com/nl/photos/aquarium-waterreservoir-4450489/<br><br>Pixabay-user: Zucky123
3	Species: Jaguar - *Panthera onca*<br>Source:<br>https://pixabay.com/nl/photos/jaguar-dier-dierentuin-natuur-1727406/<br><br>Pixabay-user: carloroberto9 (Carlo Quinteros)
4	Species: White-handed gibbon - *Hylobates lar*<br>Source:<br>https://pixabay.com/nl/photos/primaat-gibbon-withandgibbon-eten-1736282/<br><br>Pixabay-user: Kadisha (Linda)
5	Species: Fire salamander - *Salamandra salamandra*<br>Source:<br>https://pixabay.com/nl/photos/vuursalamander-salamander-dierlijke-293324/<br><br>Pixabay-user: StefanHoffmann (Stefan Hoffmann)
6	Species: American alligator - *Alligator mississippiensis*<br>Source:<br>https://pixabay.com/nl/photos/alligator-dierentuin-1335858/<br><br>Pixabay-user: JakeWilliamHeckey (Jake Heckey)
7	Species: European badger - *Meles meles*<br>Source:<br>https://pixabay.com/nl/photos/das-zoogdier-wild-natuur-fauna-4337875/<br><br>Pixabay-user: YvonneHuijbens (Yvonne Huijbens)
8	Species: Eurasian bullfinch - *Pyrrhula pyrrhula*<br>Source:<br>https://pixabay.com/nl/photos/goudvink-pyrrhula-vogel-man-boom-2450919/<br><br>Pixabay-user: Momentmal (Bernd)

9	Species: Emu - *Dromaius novaehollandiae*<br>Source:<br>https://pixabay.com/nl/photos/struisvogel-dierlijke-dierentuin-3413714/<br><br>Pixabay-user: LinArt (Lin Animalart)
10	Species: Capybara - *Hydrochoerus hydrochaeris*<br>Source:<br>https://pixabay.com/nl/photos/capibara-capybara-knaagdier-cavia-2333512/<br><br>Pixabay-user: Kapa65 (Karsten Paulick)
11	Species: Black-footed penguin - *Spheniscus demersus*<br>Source:<br>https://pixabay.com/nl/photos/pingu%C3%AFn-dierlijke-1984974/<br><br>Pixabay-user: TLSPAMG (Aniket)
12	Species: Common wombat - *Vombatus ursinus*<br>Source:<br>https://pixabay.com/nl/photos/wombat-buideldier-common-wombat-3887250/<br><br>Pixabay-user: pen_ash (Penny)
13	Species: Secretarybird - *Sagittarius serpentarius*<br>Source:<br>https://pixabay.com/nl/photos/secretarisvogel-vogel-weide-5619908/<br><br>Pixabay-user: carolabetin
14	Species: Cougar - *Puma concolor*<br>Source:<br>https://pixabay.com/nl/photos/puma-mountain-leeuw-crouching-427276/<br><br>Pixabay-user: CorinnaSt (Corinna Stoeffl)
15	Species: Red-bellied piranha - *Pygocentrus nattereri*<br>Source:<br>https://pixabay.com/nl/photos/piranha-roofvissen-vis-onderwater-5918098/<br><br>Pixabay-user: Rethinktwice

Habitat

1	Species: Meerkat - *Suricata suricatta*<br>Source:<br>https://pixabay.com/nl/photos/meerkat-dierlijke-ge%C3%AFsoleerd-4907292/<br><br>Pixabay-user: maja7777 (Jörg)
2	Species: Chamois - *Rupicapra rupicapra*<br>Source:<br>https://pixabay.com/nl/photos/chamois-hoefdieren-paarhufer-sneeuw-2053656/<br><br>Pixabay-user: strichpunkt (Nina)

3	Species: Goldcrest - *Regulus regulus*<br>Source:<br>https://pixabay.com/nl/photos/goudhaantje-regulus-regulus-vogel-2136503/<br><br>Pixabay-user: wolfgang_vogt (Wolfgang Vogt)
4	Species: Okapi - *Okapia johnstoni*<br>Source:<br>https://pixabay.com/nl/photos/okapi-dierlijke-dierentuin-zoogdier-1581236/<br><br>Pixabay-user: tenaciously_tina (Tina Robson)
5	Species: Fallow deer - *Dama dama*<br>Source:<br>https://pixabay.com/nl/photos/bos-dieren-hert-meerdere-4453874/<br><br>Pixabay-user: yvonnebridle1660
6	Species: Common crossbill - *Loxia curvirostra*<br>Source:<br>https://pixabay.com/nl/photos/curvirostra-loxia-vogel-kruisbek-387055/<br><br>Pixabay-user: PublicDomainImages
7	Species: Black-tailed prairie dog - *Cynomys ludovicianus*<br>Source:<br>https://pixabay.com/nl/photos/prairiehonden-dierentuin-4873256/<br><br>Pixabay-user: Anrita1705 (Anrita)
8	Species: Eurasian red squirrel - *Sciurus vulgaris*<br>Source:<br>https://pixabay.com/nl/photos/dierlijke-eekhoorn-possierlich-2664801/<br><br>Pixabay-user: blende12 (Gerhard G.)
9	Species: Bearded reedling - *Panurus biarmicus*<br>Source:<br>https://pixabay.com/nl/photos/vogel-bebaarde-reedling-4748669/<br><br>Pixabay-user: Psubraty (Psubraty)
10	Species: Naked mole-rat - *Heterocephalus glaber*<br>Source:<br>https://nl.wikipedia.org/wiki/Naakte_molrat#/media/Bestand:Chomez.jpg<br><br>Wikimedia commons-user: Chomez
11	Species: Southern ground hornbill - *Bucorvus leadbeateri*<br>Source:<br>https://pixabay.com/nl/photos/hoornraaf-vogel-bill-raaf-424496/<br><br>Pixabay-user: Efraimstochter (M W)
12	Species: Malayan tapir - *Tapirus indicus*<br>Source:<br>https://pixabay.com/nl/photos/maleise-tapir-asian-tapir-dierlijke-1734462/

	Pixabay-user: Coffee-king (Renato Canepa)
13	Species: Black-tailed godwit - *Limosa limosa*<br>Source:<br>https://pixabay.com/nl/photos/grutto-vogel-natuur-3648526/<br><br>Pixabay-user: kees1
14	Species: Fennec fox - *Vulpes zerda*<br>Source:<br>https://pixabay.com/nl/photos/desert-fox-dier-leuke-2444231/<br><br>Pixabay-user: hradient
15	Species: Polar bear - *Ursus maritimus*<br>Source:<br>https://pixabay.com/nl/photos/ijsbeer-roofdier-zoogdier-3468822/<br><br>Pixabay-user: maja7777 (Jörg)

Diet

1	Species: Common kingfisher - *Alcedo atthis*<br>Source:<br>https://freepngimg.com/png/160753-kingfisher-bird-beak-png-free-photo<br><br>FreePNGImg-user: Lydia Simmons
2	Species: European goldfinch - *Carduelis carduelis*<br>Source:<br>https://pixabay.com/nl/photos/vogels-putter-aard-chardonneret-4116713/<br><br>Pixabay-user: itsilatak2560
3	Species: Eurasian otter - *Lutra lutra*<br>Source:<br>https://commons.wikimedia.org/wiki/File:Lutra_lutra_2_-<br>_Otter,_Owl,_and_Wildlife_Park.jpg<br><br>Wikimedia-user: Dave Pape
4	Species: Peregrine falcon - *Falco peregrinus*<br>Source:<br>https://pixabay.com/nl/photos/vogel-slechtvalk-snavel-veren-5468788/<br><br>Pixabay-user: iulian_ursache (Iulian Ursache)
5	Species: European green woodpecker - *Picus viridis*<br>Source:<br>https://pixabay.com/nl/photos/vogel-groene-specht-boom-2970645/<br><br>Pixabay-user: JCLeroi (Alexander Lesnitsky)
6	Species: European mole - *Talpa europaea*<br>Source:

	https://pixabay.com/nl/photos/natuur-dierlijke-mole-knaagdier-13298/<br><br>Pixabay-user: Beeki (Dirk Schumacher)
7	Species: Bearded vulture - *Gypaetus barbatus*<br>Source:<br>https://pixabay.com/nl/photos/de-natuur-dieren-vogels-raptor-938467/<br><br>Pixabay-user: Inactive account – ID 422737
8	Species: Barn swallow - *Hirundo rustica*<br>Source:<br>https://pixabay.com/nl/photos/vogel-dierlijke-983941/<br><br>Pixabay-user: Free-Photos
9	Species: Common eider - *Somateria mollissima*<br>Source:<br>https://pixabay.com/nl/photos/eider-drake-zee-duikers-eenden-5940598/<br><br>Pixabay-user: Georg_Wietschorke (Georg Wietschorke)
10	Species: Koala - *Phascolarctos cinereus*<br>Source:<br>https://pixabay.com/nl/photos/dierlijke-koala-buideldier-bont-1835689/<br><br>Pixabay-user: Pexels
11	Species: Coconut lorikeet - *Trichoglossus haematodus*<br>Source:<br>https://pixabay.com/nl/photos/natuur-vogel-het-wild-levende-dieren-3134903/<br><br>Pixabay-user: Gruu (Gru)
12	Species: Marine iguana - *Amblyrhynchus cristatus*<br>Source:<br>https://pixabay.com/nl/photos/zeeleguaan-galapagos-reptiel-5949420/<br><br>Pixabay-user: eriklarsmyers (Erik Myers)
13	Species: Wolf - *Canis lupus*<br>Source:<br>https://pixabay.com/nl/photos/wolf-predator-grijze-wolf-dierlijke-3585218/<br><br>Pixabay-user: christels (Christel Sagniez)
14	Species: Gelada - *Theropithecus gelada*<br>Source:<br>https://pixabay.com/nl/photos/chelada-aap-wild-dier-9075/<br><br>Pixabay-user: extrazeit
15	Species: Giant panda - *Ailuropoda melanoleuca*<br>Source:<br>https://pixabay.com/nl/photos/panda-dierentuin-zoogdieren-pluche-4000501/<br><br>Pixabay-user: mikiange (Angelique Barillot)

Behavior

1	Species: White stork - *Ciconia Ciconia*<br>Source:<br>https://pixabay.com/nl/photos/stork-transparante-rammelaar-stork-3438129/<br><br>Pixabay-user: Inactive account – ID 2458221
2	Species: Roe deer - *Capreolus capreolus*<br>Source:<br>https://pixabay.com/nl/photos/ree-dierlijke-wilde-scheu-red-deer-1116867/<br><br>Pixabay-user: mauko (Markus Tinner)
3	Species: Red deer - *Cervus elaphus*<br>Source:<br>https://pixabay.com/nl/photos/rendier-hert-brown-dierlijke-gewei-1323000/<br><br>Pixabay-user: lagunabluemolly (Steve Mantell)
4	Species: European robin - *Erithacus rubecula*<br>Source:<br>https://pixabay.com/nl/photos/roodborstje-vogel-natuur-lente-bos-1398230/<br><br>Pixabay-user: diego_torres (Roman Grac)
5	Species: Leopard seal - *Hydrurga leptonyx*<br>Source:<br>https://pixabay.com/nl/photos/sneeuw-de-winter-koud-ijs-3368570/<br><br>Pixabay-user: jodeng
6	Species: Long-tailed tit - *Aegithalos caudatus*<br>Source:<br>https://pixabay.com/nl/photos/lange-staart-tit-vogel-kleine-vogel-5544111/<br><br>Pixabay-user: TheOtherKev (Kev)
7	Species: Common cuckoo - *Cuculus canorus*<br>Source:<br>https://pixabay.com/nl/photos/vogel-de-koekoek-veren-gevederte-5478714/<br><br>Pixabay-user: maroj10 (Manoj Ayer)
8	Species: Common shelduck - *Tadorna tadorna*<br>Source:<br>https://pixabay.com/nl/photos/bergeend-watervogel-bergeend-man-5267314/<br><br>Pixabay-user: pevank01 (Peter van Kasteren)
9	Species: African wild dog - *Lycaon pictus*<br>Source:<br>https://pixabay.com/nl/photos/zuid-afrika-wild-natuur-163056/<br><br>Pixabay-user: Yolanda (Yolanda Coervers)
10	Species: Northern pike - *Esox Lucius*<br>Source:<br>https://pixabay.com/nl/photos/snoek-nacht-onderwater-duik-vis-2276178/

	Pixabay-user: meinig (Marcel Einig)
11	Species: Grass snake - *Natrix natrix*<br>Source:<br>https://pixabay.com/nl/photos/snake-ringslang-natter-reptielen-1468685/<br><br>Pixabay-user: rihaij
12	Species: Tufted duck - *Aythya fuligula*<br>Source:<br>https://pixabay.com/nl/photos/vogels-eenden-wildlife-watervogels-3376131/<br><br>Pixabay-user: Kadisha (Linda)
13	Species: Red-backed shrike - *Lanius collurio*<br>Source:<br>https://pixabay.com/nl/photos/grauwe-klauwier-man-vogel-zittend-2550076/<br><br>Pixabay-user: LubosHouska (Lubos Houska)
14	Species: European hare - *Lepus europaeus*<br>Source:<br>https://pixabay.com/nl/photos/haas-dier-natuur-konijntje-weide-940973/<br><br>Pixabay-user: Robert_C (Robert C)
15	Species: Common warthog - *Phacochoerus africanus*<br>Source:<br>https://pixabay.com/nl/photos/knobbelzwijn-varken-wild-varken-1364424/<br><br>Pixabay-user: WiseTraveller (Richard Batka)
