# Supplementary material for: Species identification skills predict in-depth knowledge about species
Source: PLoS One. 2022 Apr 18;17(4):e0266972. doi: 10.1371/journal.pone.0266972 (PMC9015127; doi:10.1371/journal.pone.0266972)
Supplement: S1 File — (DOCX) [file pone.0266972.s004.docx]

**Odds ratios (ORs) for in-depth species knowledge among professionals and laypeople who did or did not correctly identify species, and Pearson correlations between species identification and in-depth species knowledge (subdivided into four themes).**

|  | **N** | **OR** | **95% CI** | | ***r*** | ***p*** |
| --- | --- | --- | --- | --- | --- | --- |
| **Professionals** | | | | | | |
| Origin | 920 | 9.38 | 8.56 | 10.28 | 0.73 | <0.01 |
| Habitat | 985 | 8.36 | 7.66 | 9.13 | 0.75 | <0.01 |
| Diet | 956 | 20.67 | 18.43 | 23.18 | 0.77 | <0.01 |
| Behavior | 957 | 8.51 | 7.76 | 9.33 | 0.77 | <0.01 |
| Total | 1,909 | 10.09 | 9.63 | 10.57 | 0.83 | <0.01 |
|  |  |  |  |  |  |  |
| **Laypeople** |  |  |  |  |  |  |
| Origin | 2,543 | 4.81 | 4.59 | 5.03 | 0.67 | <0.01 |
| Habitat | 2,650 | 4.94 | 4.72 | 5.16 | 0.63 | <0.01 |
| Diet | 2,681 | 13.19 | 12.46 | 13.96 | 0.70 | <0.01 |
| Behavior | 2,644 | 6.05 | 5.77 | 6.35 | 0.66 | <0.01 |
| Total | 5,259 | 6.25 | 6.10 | 6.40 | 0.76 | <0.01 |
|  |  |  |  |  |  |  |
| **All respondents** |  |  |  |  |  |  |
| Origin | 3,494 | 5.75 | 5.52 | 5.99 | 0.72 | <0.01 |
| Habitat | 3,680 | 5.72 | 5.50 | 5.95 | 0.71 | <0.01 |
| Diet | 3,675 | 15.05 | 14.31 | 15.82 | 0.76 | <0.01 |
| Behavior | 3,649 | 6.75 | 6.48 | 7.04 | 0.73 | <0.01 |
| Total | 7,249 | 7.18 | 7.04 | 7.33 | 0.81 | <0.01 |
